# ITIH4 attenuates acute lung injury by Fe-containing particulate matter in mice via Hippo pathway in type II alveolar epithelial cells

**DOI:** 10.1186/s12931-025-03256-z

**Published:** 2025-05-28

**Authors:** Vincent Laiman, Syue-Wei Peng, Lina Choridah, Didik Setyo Heriyanto, Fara Silvia Yuliani, Kang-Yun Lee, Ching-Huang Lai, Jer-Hwa Chang, Yueh-Lun Lee, Shu-Chuan Ho, Sheng-Ming Wu, Chia-Li Han, Cheng-Wei Lin, Kian Fan Chung, Hsiao-Chi Chuang

**Affiliations:** 1 Department of Radiology, Faculty of Medicine, Public Health, and Nursing, Universitas Gadjah Mada – Dr. Sardjito Hospital, Yogyakarta, Indonesia; 2 Collaboration Research Center for Precision Oncology Based Omics - PKR PrOmics, Universitas Gadjah Mada, Yogyakarta, Indonesia; 3 School of Respiratory Therapy, College of Medicine, Taipei Medical University, Taipei, Taiwan; 4 Department of Anatomical Pathology, Faculty of Medicine, Public Health, and Nursing, Universitas Gadjah Mada – Dr. Sardjito Hospital, Yogyakarta, Indonesia; 5 Department of Pharmacology and Therapy, Faculty of Medicine, Public Health, and Nursing, Universitas Gadjah Mada, Yogyakarta, Indonesia; 6 Division of Pulmonary Medicine, Department of Internal Medicine, School of Medicine, College of Medicine, Taipei Medical University, Taipei, Taiwan; 7 Division of Pulmonary Medicine, Department of Internal Medicine, Shuang Ho Hospital, Taipei Medical University, New Taipei City, Taiwan; 8 School of Public Health, National Defense Medical Center, Taipei, Taiwan; 9 Division of Pulmonary Medicine, Departments of Internal Medicine, Wan Fang Hospital, Taipei Medical University, Taipei, Taiwan; 10 Department of Microbiology and Immunology, School of Medicine, College of Medicine, Taipei Medical University, Taipei, Taiwan; 11 Master Program in Clinical Genomics and Proteomics, College of Pharmacy, Taipei Medical University, Taipei, Taiwan; 12 Graduate Institute of Medical Sciences, College of Medicine, Taipei Medical University, Taipei, Taiwan; 13 Department of Biochemistry and Molecular Cell Biology, Taipei Medical University, Taipei, Taiwan; 14 Cell Physiology and Molecular Image Research Center, Wan Fang Hospital, Taipei Medical University, Taipei, Taiwan; 15 National Heart and Lung Institute, Imperial College London, London, UK

**Keywords:** Air pollution, Autophagy, Diesel exhaust particles, Inflammation, Single-cell analysis

## Abstract

**Background:**

Metals in particulate matter (PM), like iron (Fe), were associated with lung injury. Inter-alpha-trypsin inhibitor heavy chain H4 (ITIH4) was suggested to inhibit lung inflammation. However, the effect of metals in PM, particularly Fe, on lung inflammation involving ITIH4 remained unclear.

**Methods:**

We investigated the effects of recombinant ITIH4 (rITIH4) against acute lung injury in C57BL/6JNarl and B6.*Sftpc-CreER*^*T2*^*;Ai14(RCL-tdT)-D* mice exposed to Fe-containing PM. Mice were exposed to diesel exhaust particles (DEP) or soluble iron (FeCl₃) via intratracheal instillation, while rITIH4 treatment was administered intranasally after exposure. Lung function, Fe levels (both bulk and single-cell by inductively-coupled plasma mass spectrometry (ICP-MS) and single-cell ICP-MS, respectively), inflammatory cell infiltration, and Hippo pathway regulation in type II alveolar epithelial cells (AECII) were assessed.

**Results:**

We observed correlation between lung function changes and Fe levels, both in bulk and single-cell Fe in peripheral blood mononuclear cells. Single-cell RNA sequencing of the control group identified AECII-related cells characterized by high *Sftpc, Sftpa1, Mzb1, B3 gnt5, Cacna1e,* and *Agbl1* expression. rITIH4 treatment in DEP-exposed mice restored Hippo pathway *Cdh1, Itih4, Pdpn, Wwtr1*, and *Yap1* in AECII. rITIH4 reversed DEP- and Fe-induced increases in neutrophil infiltration, neutrophil-to-lymphocyte ratio, and monocyte depletion in bronchoalveolar lavage fluid (BALF). rITIH4 reduced BALF CXCL1/KC levels by DEP and serum 8-isoprostane levels by Fe. rITIH4 also reduced DEP-induced lung damage, increased ⍺-catenin and p-YAP in Fe-exposed mice, and pTAZ/TAZ ratio in both DEP- and Fe-exposed mice. rITIH4 increased pYAP/YAP ratio in DEP-exposed mice while decreasing LC3BII/I ratio in Fe-exposed mice.

**Conclusion:**

ITIH4 attenuated acute lung injury in mice exposed to PM, specifically Fe, by modulating the Hippo pathway in AECII.

**Supplementary Information:**

The online version contains supplementary material available at 10.1186/s12931-025-03256-z.

## Background

Exposure to air pollution has been associated with increased morbidity in people with preexisting respiratory diseases [1]. Particulate matter (PM) with an aerodynamic diameter ≤ 2.5 µm (PM_2.5_) primarily originates from the combustion of fossil fuels and has been reported to induce acute lung injury, inflammation, and exacerbate respiratory diseases [2]. Additionally, PM_2.5_ can deeply infiltrate the lung, irritating and damaging the alveolar walls, leading to impaired lung function, and entering the bloodstream [3]. Health impacts of PM are particularly pronounced in people with predisposing illnesses, such as asthma, chronic obstructive pulmonary disease (COPD), and pneumonia [4]. Short-term increase of 10 μg/m^3^ in PM levels was associated with increased morbidity from cardiopulmonary conditions [5]. The pathogenicity of PM_2.5_ depends on its metal and chemical constituents, origin, and surface area to elicit adverse lung effects [3, 6]. For example, a previous study in COPD patients with ambient PM_2.5_ samples from the area of residence reported that metals like zinc (Zn), iron (Fe), and nickel (Ni) in PM_2.5_ were associated with decreased forced vital capacity (FVC) and forced expiratory volume in 1 s (FEV_1_) [6]. Fe, particularly, is essential for metabolic activities such as DNA synthesis, transcription, and mitochondrial energy production [7], which is also a major metallic compound in PM_2.5_ [8]. A previous study identified Fe (0.188 μg/m^3^) as one of the highest metals found in PM_2.5_ in Taichung City, Taiwan, further suggesting its influence on lung health upon exposure [8]. Excessive Fe can be reactive and possibly toxic resulting in programmed cell death by accumulation of lipid peroxides via the Fenton reaction [9]. Despite its significance, the mechanisms by which Fe in PM causes lung impairment remain unclear.

A previous study reported that COPD patients exposed to ambient PM exhibited unique protein profiles associated with past 3-year PM_10_ exposure levels [10]. Notably, their serum had lower levels of inter-alpha-trypsin inhibitor heavy chain H4 (ITIH4) compared to healthy controls [10]. Another study in rats exposed for 6 months to PM_2.5_ suggested that ITIH4 plays an essential role in inhibiting inflammation and alveolar epithelial cell senescence [11]. Nevertheless, there are limited studies on the effect of metals in PM, particularly Fe, on lung inflammation involving ITIH4 in the lungs.

The lungs, being the primary organs exposed to inhaled particulates, depend on epithelial cell integrity. Type II alveolar epithelial cells (AECII) serve as lung progenitors, playing a crucial role in the regeneration of functional alveoli following epithelial injury [12]. The Hippo pathway, a recently identified signaling pathway, regulates the phosphorylation of transcriptional cofactors YAP and TAZ, and activates the transcription of regeneration-related genes [13]. Recent reports have implicated the dysregulation of Hippo signaling pathway as a prominent feature in lung disease [14, 15]. A previous in vitro study on epithelial cells revealed that the adherens junction protein E-cadherin mediates contact-induced inhibition of proliferation via the Hippo signaling pathway [16]. Another study in mice with acute lung injury induced by lipopolysaccharide administration showed significantly upregulated Hippo pathway gene expressions followed by ferroptosis [17]. Limited studies have been conducted on the impact of Fe in PM on the Hippo pathway in AECII of the lung, and the potential regulatory role of ITIH4 in lung changes caused by metals, especially Fe, in PM through the Hippo pathway remains unclear. The objective of this study is to investigate the effects of ITIH4 on the Hippo signaling pathway in AECII cells regulating acute lung injury in mice exposed to PM-containing Fe. Understanding the pathways regulated by ITIH4 is crucial for elucidating the regenerative processes of AECII cells in response to metals in PM exposure.

## Materials and methods

### Animals

Male 8-week-old C57BL/6 JNarl mice were procured from the National Laboratory Animal Center (Taipei, Taiwan). The B6.129S-*Sftpc*^*tm1(cre/ERT2)Blh*^/J (Stock no: #028054) and B6.Cg-*Gt(ROSA)26Sor*^*tm14(CAG−tdTomato)Hze*^/J (Stock no: #007914) mice were obtained from The Jackson Laboratory and bred to produce B6.*Sftpc-CreER*^*T2*^*;Ai14(RCL-tdT)-D* mice in Biolasco Taiwan. Both male and female 7-week-old B6.*Sftpc-CreER*^*T2*^*;Ai14(RCL-tdT)-D* mice were used in the experiment. All mice obtained were included in the study. The temperature of the environment was 22 ± 2 ºC with relative humidity set at 55% ± 10%. The mice were housed in the laboratory animal center of Taipei Medical University with 12:12-h light: dark cycle, provided with Lab Diet 5001 (PMI Nutrition International, USA), allowed access to water ad libitum, and allowed for acclimatization. This study was conducted with the authorization of the Institutional Animal Care and Use Committee of Taipei Medical University (IACUC no. LAC-2021–0430).

### Experimental designs

Firstly, C57BL/6 JNarl mice were used to assess the metal level in peripheral blood mononuclear cells (PBMC) and evaluate changes in lung function following exposure to diesel exhaust particle (DEP) and soluble iron salt (FeCl_3_). All mice were randomized by body weight and separated into following groups, each receiving a total volume of 50 μL suspension by intratracheal administration: Control group with sterilized phosphate-buffered saline (PBS); DEP group with 8.5 mg/kg of DEP suspension (SRM 2975, NIST, Gaithersburg, MD, USA) as referenced from the previous study to induce substantial acute lung injury [18]; and FeCl_3_ group with 0.05 mg/kg of FeCl_3_ suspension (Sigma-Aldrich, St. Louis, MO, USA) based on the equivalent concentration of Fe (1.25 ng/µg) in DEP [18, 19]. The DEP has been characterized previously, with pore diameters ranging from 4 to 35 nm and most particles measuring around 20 nm [20]. Chemical characterization of DEP has also been studied, with the majority being carbon, nitrogen, oxygen, sulfur, copper (Cu), and Zn [19, 20]. Exposure by intratracheal administration was performed under general anesthesia maintained by 3% isoflurane using anesthesia machine for rodent (Northern Vaporisers; Skipton, UK). Secondly, B6.*Sftpc-CreER*^*T2*^*;Ai14(RCL-tdT)-D* mice were used and pre-treated with Tamoxifen (Sigma, St. Louis, MO, USA), dissolved in 99.8% ethanol (Honeywell, Charlotte, NC, USA) and corn oil (Sigma, St. Louis, MO, USA) with final concentration of 12.5 mg/mL, by intraperitoneal injection at concentration of 62.5 mg/kg/day for 5 days to induce Cre-recombinase before the experiment begun. The mice were then separated into 6 groups: Control (PBS), Control + rITIH4, DEP, DEP + rITIH4, FeCl_3_, and FeCl_3_ + rITIH4. Mice were first exposed to either PBS, DEP, or FeCl_3_. One hour after exposure, the mice were intranasally administered with 50 µL of either sterilized PBS or 12.5 µg/kg recombinant human ITIH4 (rITIH4) (CSB-EP617923HU, Cusabio, Houston, TX, USA) [21, 22]. After 24 h., lung function tests were performed, and mice were sacrificed.

### Lung functions examination

The lung functions were measured by Flexivent (SCIREQ; Sterling, VA, US). The mice were anesthetized, instilled with soft catheter of 24-gauge through the trachea, and connected with the ventilator in Flexivent. Details of the lung functions examination were described in section S1 in supplementary information (SI).

### Bronchoalveolar lavage (BALF) differential cell count

The BALF was obtained from the mice and cell counting in the BALF was performed by hematology analysis (ProCyte Dx; IDEXX Laboratories; Westbrook, Maine, USA). The numbers of neutrophils, eosinophil, lymphocytes, and monocytes were obtained and reported as percentages of total white blood cell counts. The neutrophil-to-lymphocyte ratio (NLR) and eosinophil-to-lymphocyte ratio (ELR) were determined as indicators of subclinical inflammation [23]. The BALF collection and processing were detailed in section S2 in SI.

### Serum and PBMC collection

Serum and PBMC sample were collected from each mouse after treatment. PBMC was fixed with 4% paraformaldehyde. The serum and PBMC collection and processing procedures were detailed in Section S3 of the SI.

### Metals in PBMC determined by bulk inductively coupled plasma mass spectrometry (ICP-MS)

PBMC was used for analysis of bulk metal contents by ICP-MS using iCAP RQ ICP-MS spectrometer (Thermo Fisher Scientific, MA, USA). The ICP-MS was used to determine twelve metal concentrations: Arsenic (As), Cadmium (Cd), Cobalt (Co), Chromium (Cr), Cu, Fe, Manganese (Mn), Ni, Lead (Pb), Selenium (Se), Vanadium (V), and Zn. Details of the bulk ICP-MS methodology were described in section S4 of SI.

### Single-cell Fe in PBMC determined by single-cell (sc) ICP-MS

PBMC sample was used for identification of single-cell Fe (scFe) content using scICP-MS. Deionized water blanks and 56 Fe (12,310–7439-89–6, Sigma, St. Louis, MO, USA) was used as standard for Fe content in cell determination. Details of the scICP-MS methodology were described in section S5 of SI.

### Single-cell RNA sequencing (scRNA-Seq)

Whole lung sample was collected from one mouse per group (control, DEP, and DEP + ITIH4; *n* = 1 per group), and the lung was perfused to eliminate red blood cells. Lung dissociation was performed, and cell pellets were collected. Cell pellets were analyzed using 10 × Single Cell 3’ v3 sequencing kit (10X Genomics, Pleasanton, CA, USA) following the protocol provided by the company. Cell prefiltering was performed following quality control criteria including 500–4000 expressed genes, less than 20% of UMIs mapped to mitochondrial genes, and less than 20,000 total UMIs. Automatic annotation on cell types of clusters from single-cell RNA sequencing data was performed using scCATCH [24]. Loupe™ Cell Browser v2.1 (10X Genomics) was used to perform analysis in gene expressions in AECII *Epcam* positive (*Epcam*^+^) and *Sftpc* positive (*Sftpc*^+^) cells. Details of the scRNA-Seq analysis were described in section S6 of SI.

### Protein extraction in lung tissue

The cell lysis reagent CelLytic MT (Sigma, St. Louis, MO, USA) with 1% ethylenediaminetetraacetic acid and 1% protease were used for lung tissue proteins extraction by homogenizing lung tissues with tissue grinder. Samples were centrifuged at 16,000 g in 4ºC centrifuge for 30 min. Supernatants were subsequently collected and kept at −80 °C for later use.

### Enzyme-linked immunosorbent assay (ELISA)

Levels of interleukin (IL)−1β (432,604, BioLegend, San Diego, CA, USA), chemokine (C-X-C motif) ligand 1(CXCL1)/keratinocyte chemoattractant (KC) (447,504, BioLegend, San Diego, CA, USA), 8-isoprostane (516,351, Cayman, Ann Arbor, MI, USA), and ITIH4 (SEH776Mu, Cloud-Clone Corp., Wuhan, China) were determined in BALF and serum by ELISA according to the manufacturer’s instructions.

### Western blotting

Protein concentrations of the lung lysates were measured using the BCA Protein Assay Reagent Kit (Bio-Rad, Hercules, CA, USA). Protein extracts were then transferred to a membrane (polyvinylidene difluoride blotting membranes) (PerkinElmer, Waltham, MA, USA). Blocking of the blots was performed followed by incubation with the primary antibody and horse radish peroxidase-labelled secondary antibody. The ChemiDoc™ MP Imaging System (Bio-Rad, CA, USA) was used to obtain image and Image-Pro vers. 4 (Media Cybernetics, Inc., MD, USA) for Windows was used to quantify the expression. The data were quantified and normalized to the control group, with expression levels presented as fold change relative to the control. The western blot methods and antibodies used were described further in section S7 of SI.

### Lung damage assessment

Lung sections were fixed using 10% buffered formalin by tracheal instillation at a pressure of 25 cmH_2_O for 10 min. Following that, lung tissues were paraffin embedded, sectioned, and stained with hematoxylin and eosin (H&E). Motic Easyscan Pro and Motic DSAssistant software (Motic, Xiamen, Fujian, China) was used to obtain lung H&E images. Assessment of lung damage was conducted by using K-means clustering algorithm by ImageJ software (National Institute of Health, Bethesda, MD, USA) [25, 26]. The areas of maximal damage (red) were identified as Severe zones, the areas of active morphological remodeling (green) as Mild zones, and the homeostatic appearing regions (blue) as Normal zones. The lung damage assessment method was detailed in Section S8 of the SI.

### Immunofluorescence (IF) staining

For lung sections of B6.*Sftpc-CreER*^*T2*^*;Ai14(RCL-tdT)-D* mice, lungs were perfused with 4% paraformaldehyde for 4 h., submerged in 30% sucrose solution overnight, and then frozen in optimal cutting temperature compound (Leica, Deer Park, IL, USA) over liquid nitrogen before being sectioned using cryostat (CM3050S, Leica, Deer Park, IL, USA). Sections then underwent blocking step using a 5% bovine serum albumin (BSA) solution followed by incubation with primary and secondary antibody. Fluorescent images were captured at 20 × magnification using a fluorescence microscope (Echo Revolve, Echo, San Diego, CA, USA). Details of the immunofluorescence staining protocol and antibodies used were detailed in section S9 of SI.

### Statistical analysis

Data were expressed as the mean ± SD. For multiple comparisons, analysis of variance (ANOVA) with post-hoc Tukey test was used. Spearman’s correlation coefficients were used to examine correlations of lung function with metals in PBMC. Visualization of Spearman’s correlation was done with ggplot2 package by RStudio (vers. 4.1.1) for macOS. Statistical analyses and quantitative figures were performed using GraphPad v. 9 (San Diego, CA, USA) for macOS. The level of significance was set to *p* < 0.05.

## Results

### Fe and scFe levels in PBMC were correlated with lung function changes

**Fig. 1:**
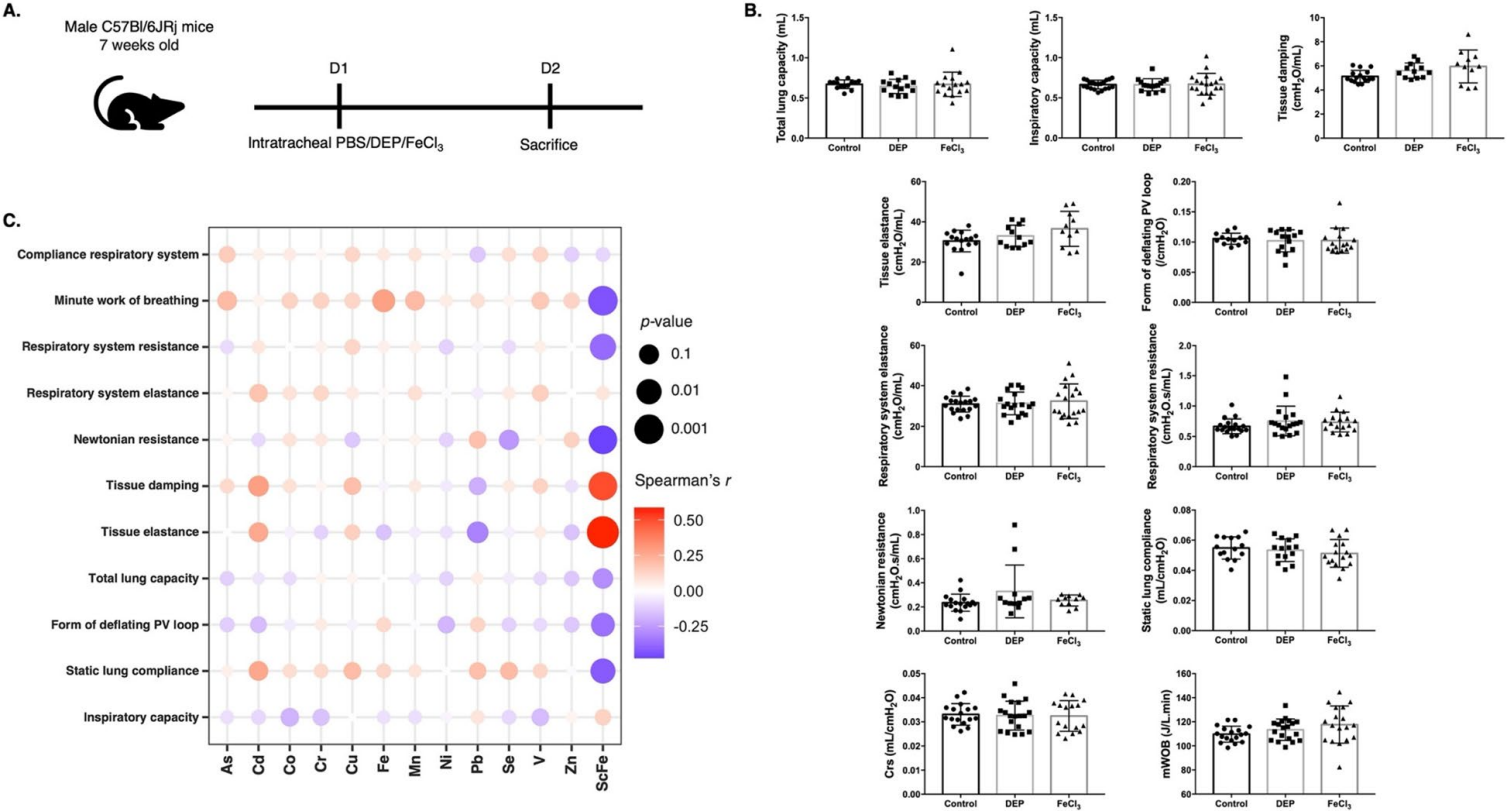
**A** Schematic diagram of experiment in which male C57Bl/6 JNarl mice of 8 weeks old were intratracheally administered with either phosphate buffered saline (PBS), diesel exhaust particle (DEP), and soluble iron salt (FeCl_3_). The experiment was concluded on day 2. **B** Lung function tests in C57Bl/6 JNarl mice in control, DEP, and FeCl_3_ groups (*n* = 14–18). **C** Correlation heatmap between lung function and metals in peripheral blood mononuclear cells. The color depth represents the strength of the correlation coefficient (red: positive correlation; blue: negative correlation). The size of the data point reflects the significance of the correlation. CRS, compliance respiratory system; mWOB, minute work of breathing; scFe, single-cell Fe

C57BL/6 JNarl mice were exposed to DEP and FeCl_3_ (Fig. 1A). We observed that the three highest bulk metals identified in PBMC were Cu, Zn, and Fe (Table 1). Figure 1B shows no significant changes in lung function after exposure to DEP and FeCl_3_. Correlation analysis showed that Fe level in PBMC was positively correlated with minute work of breathing (mWOB) (*r* = 0.285, *p* < 0.05) (Fig. 1C). The scFe in PBMC was negatively correlated with static lung compliance (*r* = −0.417, *p* < 0.05), form of deflating PV loop (*r* = −0.360, *p* < 0.05), Newtonian resistance (*r* = −0.475, *p* < 0.05), respiratory system resistance (*r* = −0.376, *p* < 0.05), and mWOB (*r* = −0.436, *p* < 0.05). The scFe was also positively correlated with tissue elastance (*r* = 0.586, *p* < 0.05) and tissue damping (*r* = 0.498, *p* < 0.05).

**Table 1:** Metal concentrations in the peripheral blood mononuclear cells of C57Bl/6 JNarl mice among control, diesel exhaust particle (DEP), and soluble iron (FeCl_3_) groups

	Control	DEP	FeCl_3_
As (ppb)	0.04 ± 0.05	0.04 ± 0.04	0.04 ± 0.05
Cd (ppb)	0.15 ± 0.19	0.09 ± 0.16	0.09 ± 0.13
Co (ppb)	0.02 ± 0.02	0.02 ± 0.03	0.02 ± 0.02
Cr (ppb)	0.38 ± 0.31	0.33 ± 0.3	0.45 ± 0.6
Cu (ppb)	39.13 ± 24.69	30.07 ± 22.48	37.45 ± 21.67
Fe (ppb)	7.01 ± 7.5	7.07 ± 10.15	6.75 ± 9.36
Mn (ppb)	0.95 ± 0.77	0.96 ± 0.85	0.89 ± 0.78
Ni (ppb)	3.83 ± 2.77	3.79 ± 3.37	5 ± 5.13
Pb (ppb)	1.18 ± 0.92	0.78 ± 0.28	0.99 ± 0.64
Se (ppb)	0.17 ± 0.17	0.16 ± 0.23	0.16 ± 0.22
V (ppb)	0.06 ± 0.04	0.06 ± 0.03	0.07 ± 0.05
Zn (ppb)	20.03 ± 10.22	25.33 ± 16.79	26.08 ± 16.86
scFe (fg/cell)	630.58 ± 441.58	503.84 ± 471.62	558.93 ± 382.02

*Abbreviations: As* Arsenic, *Cd* Cadmium, *Co* Cobalt, *Cr* Chromium, *Cu* Copper, *Fe* Iron, *Mn* Manganese, *Ni* Nickel, *Pb* Lead, *Se* Selenium, *V* Vanadium, *Zn* Zinc, *scFe* Single-cell Fe

### ITIH4 involved in Hippo pathway-related gene expressions in AECII by DEP exposure

**Fig. 2:**
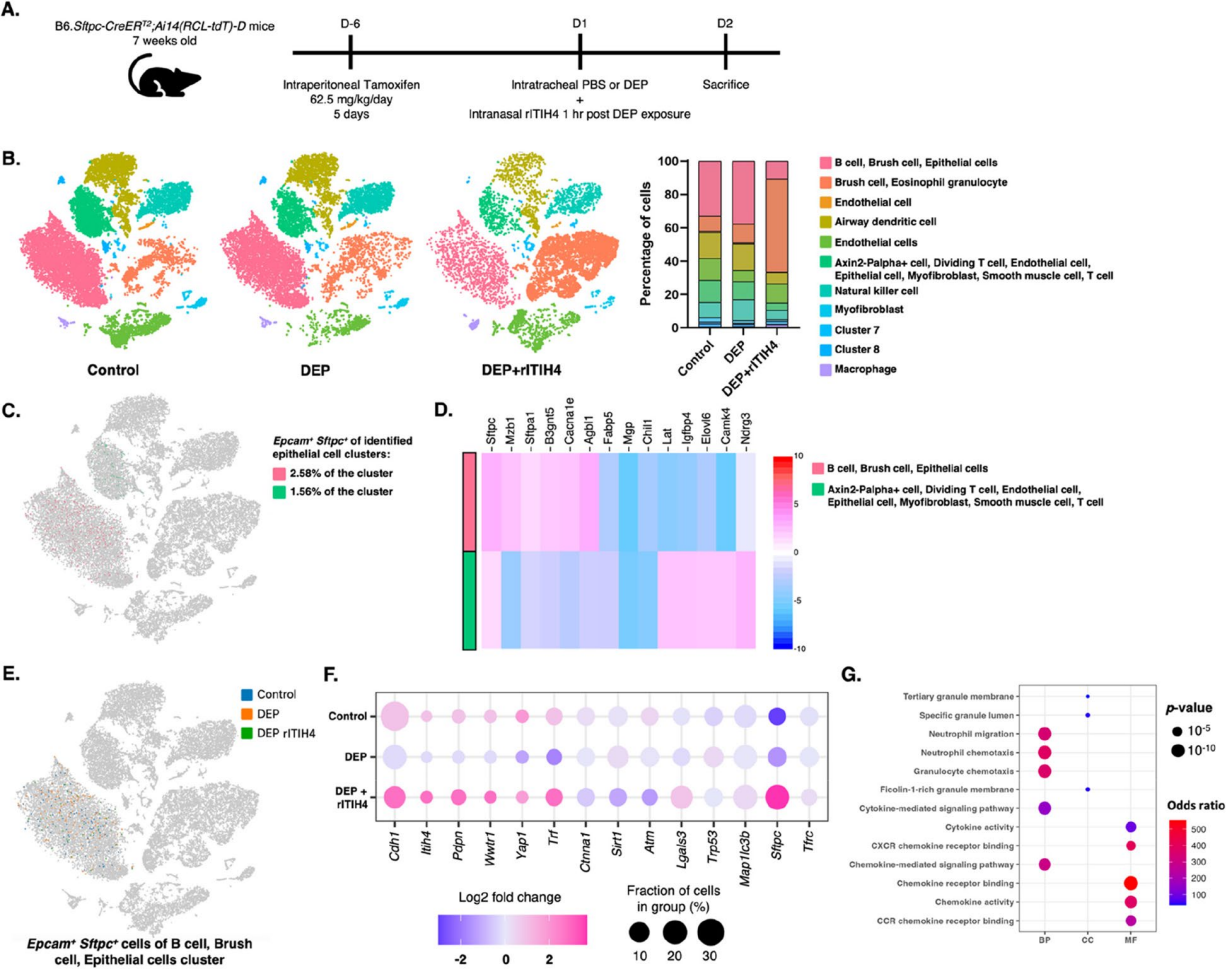
**A** Schematic diagram of 7 weeks old B6*.Sftpc-CreER*^*T2*^*;Ai14(RCL-tdT)-D* mice pre-treated with Tamoxifen injection were intratracheally administered with either phosphate buffered saline (PBS) or diesel exhaust particle (DEP). After 1 h, the DEP-exposed mice were intranasally administered with recombinant ITIH4 (rITIH4) protein. **B** t-stochastic neighbor embedding (t-SNE) plot of all cells colored by their cellular identity and percentages of each cells cluster in lung of B6*.Sftpc-CreER*^*T2*^*;Ai14(RCL-tdT)-D* mice in control, DEP, and DEP with rITIH4 treatment groups. **C** t-SNE plot, percentages of cells, and (**D**) heatmap displaying the gene expressions of type II alveolar epithelial cell (AECII) by *Epcam*-positive (*Epcam*^+^) and *Sftpc*-positive (*Sftpc*^+^) cells within the B cell, brush cell, and epithelial cell cluster (pink), as well as the Axin2-Palpha + cell, dividing T cell, endothelial cell, epithelial cell, myofibroblast, smooth muscle cell, and T cell cluster (green). **E** t-SNE plot of AECII cells (*Epcam*^+^ *Sftpc*^+^) within the B cell, brush cell, and epithelial cell cluster and (**F**) dot plot of gene expressions related to Hippo pathway in control, DEP, and DEP with rITIH4 treatment groups. The depth of the color indicates log2 fold changes (red: positive fold change; blue: negative fold change). The size of the point indicates the percentage of cells in the group. **G** Gene ontology enrichment analysis related to biological processes (BP), cellular component (CC), and molecular function (MF). The depth of the color indicates the activation odds ratio. The size of the point indicates the *p*-values of the enrichment analysis

To understand the Hippo pathway regulation in lung AECII, scRNA-Seq was conducted in lung of B6.*Sftpc-CreER*^*T2*^*;Ai14(RCL-tdT)-D* mice with DEP exposure and rITIH4 treatment (Fig. 2A)*.* We classified cells into 11 groups of cell types, which included 9 clusters of identified multiple cells (B cell, brush cells, epithelial cells, eosinophil granulocytes, endothelial cells, airway dendritic cells, T cells, myofibroblasts, smooth muscle cells, natural killer cells, and macrophages) and 2 distinct unknown cluster (Fig. 2B). We observed dynamic changes in the cellular composition and percentage of cells in each cluster of lungs between control, DEP, and DEP with rITIH4 treatment. The B cells, brush cells, and epithelial cells cluster occupied higher proportions in both control (33.1%) and DEP (37.8%) groups. However, brush cells and eosinophil granulocytes cluster was the most abundant cell population in DEP + rITIH4 (55.9%) group. We identified the AECII progenitor cells (*Epcam*^+^ *Sftpc*^+^ cells) in the first cluster (B cell, brush cell, and epithelial cell) and second cluster (Axin2 P-alpha^+^ cell, dividing T cell, endothelial cell, epithelial cell, myofibroblast, smooth muscle cell, and T cell) and found that the clusters consisted of 2.58% and 1.56% of AECII cells, respectively (Fig. 2C). Further analysis in these epithelial cells clusters showed that the first cluster exhibited higher expressions of AECII-related genes, including *Sftpc, Sftpa1, Mzb1, B3 gnt5, Cacna1e*, and *Agbl1*, while the second cluster showed higher expressions of *Lat, Igfbp4, Elovl6, Camk4*, and *Ndrg3* (Fig. 2D). We then selected the AECII cells in the first cluster and investigated the Hippo pathway components between control, DEP, and DEP with rITIH4 treatment groups (Fig. 2E). We found that five genes (*Cdh1, Itih4, Pdpn, Wwtr1,* and *Yap1*) were downregulated by DEP exposure and improved following ITIH4 administration (Fig. 2F). Gene ontology (GO) analysis revealed enrichment in neutrophil migration, neutrophil and granulocyte chemotaxis, and chemokine-related signaling pathway and binding following rITIH4 treatment (Fig. 2G).

### ITIH4 reduced lung inflammation, oxidative stress, and lung damage

**Fig. 3:**
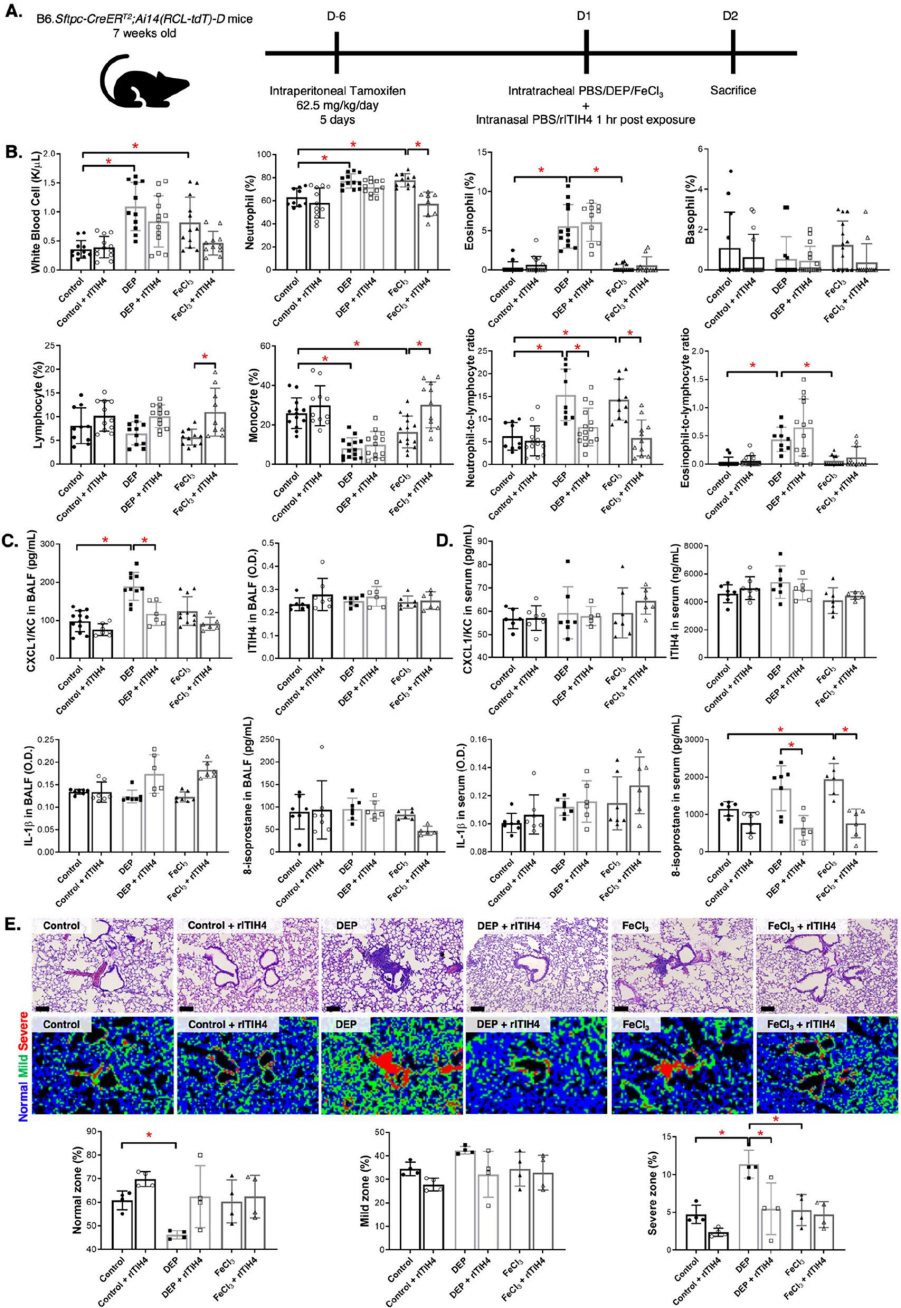
**A** Schematic diagram of 7 weeks old B6*.Sftpc-CreER*^*T2*^*;Ai14(RCL-tdT)-D* mice pre-treated with Tamoxifen injection were intratracheally administered with either phosphate buffered saline (PBS), diesel exhaust particle (DEP), or soluble iron salt (FeCl_3_). After 1 h, the mice were intranasally administered with either PBS or recombinant ITIH4 (rITIH4) protein. **B** Inflammatory cells differential count in bronchoalveolar lavage fluid (BALF) of B6*.Sftpc-CreER*.^*T2*^*;Ai14(RCL-tdT)-D* mice in control, control with rITIH4, DEP, DEP with rITIH4, FeCl_3_, and FeCl_3_ with rITIH4 groups (*n* = 8–12) (**C**) Measurement of chemokine (C-X-C motif) ligand 1(CXCL1)/keratinocyte-derived chemokine (KC), ITIH4, interleukin (IL)−1β, and 8-isoprostane in BALF and (**D**) CXCL1/KC, rITIH4, IL-1β, and 8-isoprostane in serum of mice in control, control with rITIH4, DEP, DEP with rITIH4, FeCl_3_, and FeCl_3_ with rITIH4 groups (*n* = 6–11). *p < 0.05. **(E)** Lung damage assessment of mice in control, control with rITIH4, DEP, DEP with rITIH4, FeCl_3_, and FeCl_3_ with rITIH4 groups (scale bar = 100 μm). The percentages of normal zone indicating homeostatic appearing areas (blue), mild zone indicating active morphological remodeling areas (green) as, and severe zone indicating areas of maximal damage (red) were shown (*n* = 4). *p < 0.05

To understand the effect of ITIH4 on resolving lung injury, B6.*Sftpc-CreER*^*T2*^*;Ai14(RCL-tdT)-D* mice were exposed to DEP or FeCl_3_ then treated with rITIH4 (Fig. 3A). Both DEP and FeCl_3_ exposure significantly increased the total white blood cells in the BALF (*p* < 0.05) (Fig. 3B). We observed significant increase in neutrophil and significant decrease in monocyte in mice with DEP and FeCl_3_ exposure (*p* < 0.05). The increase in neutrophil and decrease in monocyte were significantly reversed in FeCl_3_ groups by rITIH treatment (*p* < 0.05). The eosinophils and ELR were significantly increased by DEP compared to both control and FeCl_3_ groups (*p* < 0.05). The NLR was significantly increased by DEP and FeCl_3_, which was reduced by rITIH4 treatment (*p* < 0.05). The CXCL1/KC in BALF was significantly increased by DEP, but decreased by rITIH4 administration (*p* < 0.05) (Fig. 3C). The 8-isoprostane in serum was significantly increased by FeCl_3_ group, but decreased by rITIH4 (*p* < 0.05) (Fig. 3D). DEP exposure significantly decreased the normal zone of lung damage compared to control group (*p* < 0.05) (Fig. 3E). We also observed that severe zone of lung damage was significantly increased by DEP, which was decreased by rITIH4 (*p* < 0.05).

### ITIH4 increased cell adhesion and YAP/TAZ phosphorylation

**Fig. 4:**
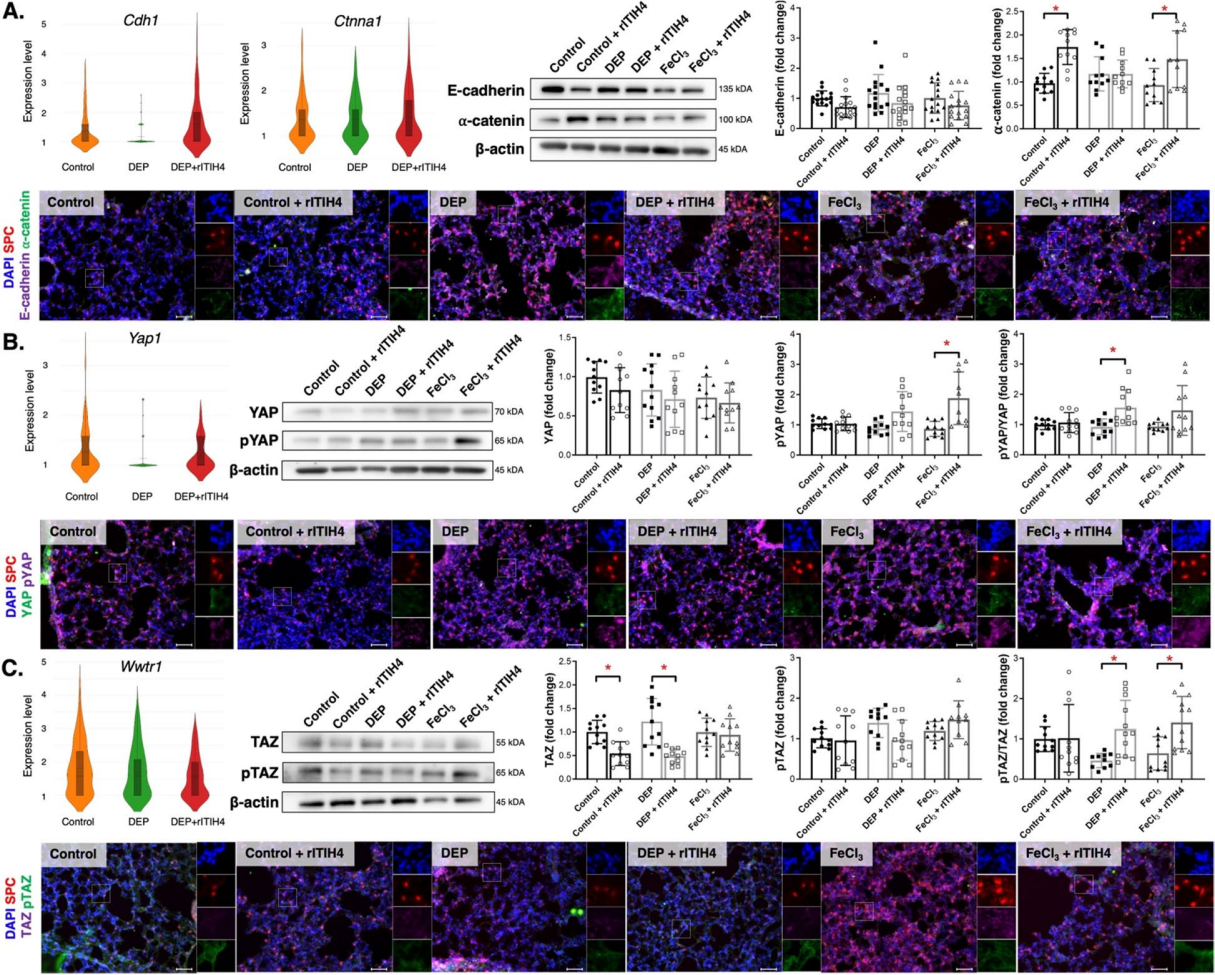
Violin plot of gene expressions (presented in log2 fold changes) in type II alveolar epithelial cells (AECII) *Epcam*^+^ *Sftpc*^+^ cells by single-cell RNA in lungs of B6*.Sftpc-CreER*^*T2*^*;Ai14(RCL-tdT)-D* mice in control, diesel exhaust particle (DEP), and DEP with rITIH4, representative western blot images, quantitative analysis of the western blot images with normalization to β-actin compared to control group, and representative immunofluorescent staining of lungs of mice in control, control with rITIH4, DEP, DEP with rITIH4, soluble iron (FeCl_3_), and FeCl_3_ with rITIH4 groups. **A** Violin plot of *Cdh1* and *Ctnna1*, western blot images of E-cadherin and ⍺-catenin, and immunofluorescent staining with SPC (red), E-cadherin (purple), and ⍺-catenin (green) (nuclear staining with DAPI in blue). **B** Violin plot of *Yap1*, western blot images of YAP and p-YAP, and immunofluorescent staining with SPC (red), YAP (green), and p-YAP (purple) (nuclear staining with DAPI in blue). **C** Violin plot of *Wwtr1*, western blot images of TAZ and p-TAZ, and immunofluorescent staining with SPC (red), TAZ (purple), and p-TAZ (green) (nuclear staining with DAPI in blue) (scale bar = 50 μm). ^*^p < 0.05

Violin plot of gene expression showed that *Cdh1* expression in AECII was clearly decreased in DEP group and reversed by rITIH4 treatment (Fig. 4A). We observed that ⍺-catenin was increased by rITIH4 administration in the control and FeCl_3_ groups (*p* < 0.05). The ⍺-catenin in SPC^+^ cell was increased in FeCl_3_ + rITIH4 mice compared to FeCl_3_ mice group. The *Yap1* expression in AECII was decreased in DEP group and increased by rITIH4 treatment (Fig. 4B). The pYAP and pYAP in SPC^+^ cell was increased in FeCl_3_ + rITIH4 compared to FeCl_3_ group, with the pYAP/YAP ratio significantly increased in DEP + rITIH4 compared to DEP group (*p* < 0.05). rITIH4 treatment slightly decreased the *Wwtr1* expression in AECII (Fig. 4C). Treatment with rITIH4 significantly decreased the TAZ expression in control and DEP groups while increasing pTAZ/TAZ ratio in both DEP and FeCl_3_ groups (*p* < 0.05).

### Reduction of autophagy by ITIH4

**Fig. 5:**
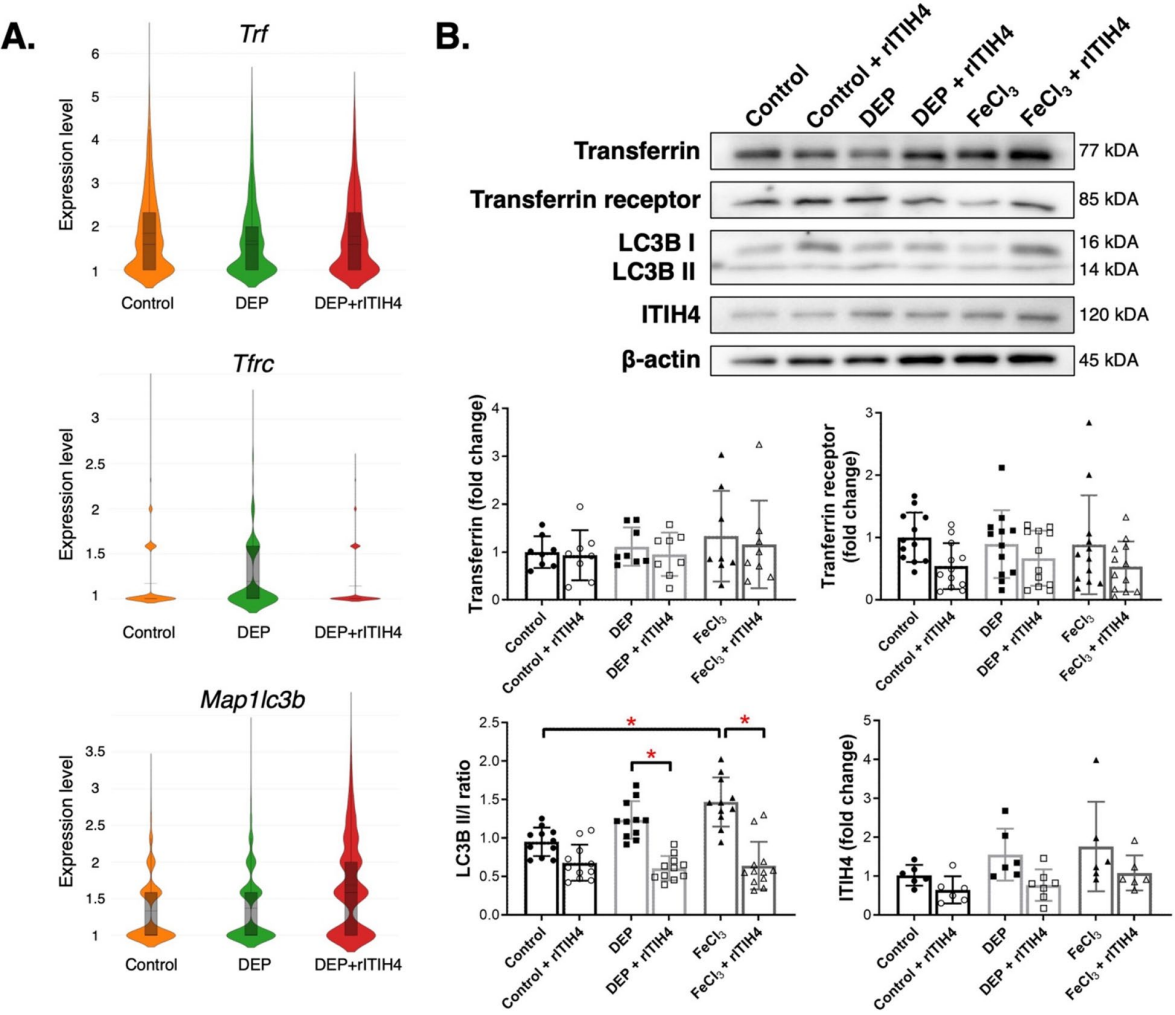
**A** Violin plots (presented in log2 fold changes) of *Trf*, *Trfc*, and *Map1 lc3b* in type II alveolar epithelial cells (AECII) *Epcam*^+^ *Sftpc*^+^ cells by single-cell RNA of B6*.Sftpc-CreER*^*T2*^*;Ai14(RCL-tdT)-D* mice lungs in control, diesel exhaust particle (DEP), and DEP with recombinant ITIH4 (rITIH4). **B** representative western blot images and quantitative analysis of the western blot images with normalization to β-actin compared to control group, and representative immunofluorescent staining of lungs of mice in control, control with rITIH4, DEP, DEP with rITIH4, soluble iron (FeCl_3_), and FeCl_3_ with rITIH4 groups. ^*^p < 0.05

Violin plots depicting AECII gene expressions showed elevated *Tfrc* level in the DEP group compared to both control and FeCl_3_ groups while *Map1 lc3b* expression was increased in DEP + rITIH4 group (Fig. 5A). We observed that FeCl_3_ exposure significantly increased the LC3BII/I ratio compared to control (Fig. 5B) (*p* < 0.05). We also found that treatment of rITIH4 significantly decreased the LC3BII/I ratio in both DEP and FeCl_3_ groups (*p* < 0.05).

## Discussion

The novelty of this study is that we investigated the effects of rITIH4 administration on acute lung injury and the underlying mechanisms in AECII by DEP and Fe exposure. The main findings of this study were that: (1) ITIH4 restored AECII Hippo pathway-related gene changes by DEP exposure, and (2) ITIH4 decreased lung injury, increased cell adhesion protein, YAP/TAZ phosphorylation, and decreased autophagy. These highlight ITIH4's therapeutic potential in mitigating the adverse effects of PM on lung by modulating the Hippo signaling pathway in AECII.

We observed correlations between the scFe in PBMC and changes in lung functions, consistent with a prior study on systemic factors affecting lung health [27]. The study reported that median levels of serum ferritin correlated with the degree of airway obstruction and smoking exposure [27]. PM_2.5_ consisted of metals, with Fe being a major component [8, 28]. DEP had been extensively studied and is commonly used to represent traffic-related air pollution [19]. Therefore, we determined the DEP dose sufficient to induce oxidative stress and inflammation, as observed in air pollution-related pathomechanisms [29, 30]. Furthermore, we administered Fe to represent the metals found in traffic-related PM, using an equivalent concentration of Fe (1.25 ng/µg) present in DEP [18, 19]. Nevertheless, despite observing correlations between lung functions with Fe and scFe levels in PBMC, we did not observe significant changes in lung functions among the control, DEP, and FeCl_3_ groups, suggesting potential threshold effects in the regulation of lung function by PM exposure. These underscore the need for further investigation into the molecular interplay involving Fe and changes in lung function.

Next, we identified AECII by scRNA with high expression of *Sftpc, Sftpa1, Mzb1, B3 gnt5, Cacna1e*, and *Agbl1* in response to DEP exposure. The *Sftpc* and *Sftpa1* were well-established AECII marker, with increased *B3 gnt5* expression in A549 lung epithelial cells enhancing cell proliferation and colony formation [12, 31]. The *Mzb1* played an important role in antibody secretion while polymorphism of rs4513061 in *Agbl1* was linked to lower lung cancer risk [32, 33]. Interestingly, higher *Cacna1e* expression was reported in patients with long-term exposure to household air pollution, suggesting its involvement in air pollution-related AECII effects [34]. Together, these findings support that AECII were involved in activation of genes related to cell proliferation and PM of air pollution.

Notably, we found that rITIH4 restored Hippo pathway components in AECII progenitor cells exposed to DEP. In a previous study, single-dose lipopolysaccharide injury in lungs of mice prevented AECII proliferation and type I alveolar epithelial cells (AECI) differentiation, with YAP/TAZ activity in Hippo pathway involved in alveolar regeneration [35]. On the other hand, ITIH4 promoted epithelial adhesion and migration, suggesting its potential role in repairing lung injury [36]. Our previous study on traffic-related air pollution-exposed rats for 6 months also suggested that ITIH4 might be a specific protein responding to air pollution in the alveolar epithelium [11]. This finding was supported by a previous study in COPD patients showing the relationship between ITIH4 and PM [10]. Therefore, we administered rITIH4 to DEP-exposed mice in our study, and our findings aligned with those previous studies. Despite DEP exposure causing cell composition changes, rITIH4 improved Hippo pathway components in AECII lung progenitor cells, indicating its important role in genes improving genes related to lung restoration. Additionally, we found that rITIH4 administration enriched neutrophil and granulocyte chemotaxis, as well as chemokine-related signaling pathway and binding-related gene expressions. A previous study also showed that ITIH-deficient mice had increased complement binding and phagocytosis, suggesting activation of chemokine-related gene expressions leading to inflammation and lung injury [37]. Our previous study also reported ITIH4 increased E-cadherin and β-catenin cell adherens junction proteins in AECII exposed to lipopolysaccharide-induced inflammation [21]. We have also reported that cell adherens junctions serve as upstream regulators of YAP and TAZ in cellular regulation [15]. Confirming this, we found that rITIH4 increased cell adhesion protein and YAP/TAZ phosphorylation. The Hippo pathway regulates organ development, homeostasis, and regeneration, cell proliferation, differentiation, and spatial patterning in organ development and injury-induced regeneration [39]. A previous study also reported increased AECII TAZ phosphorylation as the process of AECII activation and differentiation after TAZ signal activation [40]. The increased YAP/TAZ phosphorylation observed in our study therefore suggests differentiation of AECII by ITIH4. Taken together, our results suggest that rITIH4 administration could restore cell composition changes, Hippo signaling pathway activity, and chemokine-related gene expression changes in AECII exposed to DEP.

We observed an increase in lymphocyte percentages and monocyte levels following rITIH4 treatment, further emphasizing its potential immunomodulatory effects. These aligned with a previous study on the anti-inflammatory properties of ITIH4 and its role in modulating immune responses [36]. The significant increase in CXCL1/KC levels in BALF following DEP administration also aligned with a previous study on the pro-inflammatory effects of PM exposure in the lung [41]. We also observed a subsequent decrease in CXCL1/KC following rITIH4 treatment, which suggested its potential role in mitigating the inflammatory response induced by DEP. Moreover, the increase in 8-isoprostane levels in the FeCl_3_ group underscored the potential role of iron in oxidative stress and its implications for lung health [42]. Interestingly, we observed a reduction in 8-isoprostane levels following rITIH4 treatment in both DEP- and FeCl₃-exposed groups. ITIH4 likely mitigated oxidative stress, one of the key mechanisms by which PM induced lung injury, through specific functional groups or domains that modulated redox balance or inflammatory signaling, resulting in a significant decrease in 8-isoprostane levels. While DEP exposure also triggers oxidative stress and inflammation, its complex and variable composition (e.g., organic and inorganic chemicals and metals) induced distinct molecular pathways and may not be effectively regulated by ITIH4 [19, 43, 44]. For instance, polycyclic aromatic hydrocarbons in DEP exposure could lead to downstream effects such as increased CYP1 A1 and HO-1 gene expression, MAPK activation, and even DNA damage via p53 and Chk1/Chk2 phosphorylation [45, 46]. Additionally, DEP has been widely used to study traffic-related air pollution, but studies have shown that DEP's biological impact can vary depending on its source and composition [15, 19, 47]. One study reported TRPA1 activation across several types of DEP in A549 and IMR-90 lung cells, but only specific variants triggered IL-8 production which suggested that the effects of DEP were shaped by its physicochemical diversity [47]. On the other hand, Fe exposure primarily drives inflammation through reactive oxygen species generation, triggering pathways such as the hypoxia-inducible factor (HIF) signaling and ferroptosis. These pathways were more directly related to oxidative stress and iron dysregulation—mechanisms in which ITIH4 may play a regulatory role. Furthermore, in this study, the dose of FeCl₃ used was selected to be equivalent to the Fe content found in DEP (1.25 ng/µg) [18, 19], allowing us to study the specific contribution of Fe to the observed effects. Therefore, while DEP exposure modeled the overall impact of PM in the lung, FeCl₃ served as a more targeted approach to study the impact of Fe related to DEP, which may better reflect the pathways modulated by ITIH4. This finding highlighted the potential antioxidative role of ITIH4 in reducing oxidative stress in the lungs caused by PM, which may partially contribute to its anti-inflammatory effects. Confirming the changes in cytokine and oxidative stress, we also observed that DEP exposure increased lung damage severity, while rITIH4 treatment decreased DEP-induced lung damage severity. A previous study with DEP exposure by intratracheal instillation in mice reported increased neutrophils, eosinophils, and lung injury [48]. Additionally, increased activity of nicotinamide adenine dinucleotide phosphate (NADPH) cytochrome P‐450 superoxide producer and decreased activities of superoxide scavengers were suggested to be implicated in DEP-induced lung damage [43]. Interestingly, the improvement in lung damage severity by ITIH4 had been reported previously on deficient circulating ITIH mice, demonstrating that ITIH attenuated complement-induced lung injury, suggesting a protective role of ITIH in lung [37]. Together, these findings aligned with our observation that decreased cytokine levels by rITIH4 were associated with reduced lung damage, suggesting that complement inhibition may be a possible function of ITIH4 in PM-induced lung damage.

We also observed that FeCl_3_ exposure increased autophagy, while treatment with rITIH4 decreased autophagy in both the DEP and FeCl_3_ groups. The injury by FeCl_3_ exposure was potentiated by reactive iron (Fe^2+^) leading to oxidative stress and lysosomal leakage, which then initiates cellular reparative processes, enhancing autophagy [49]. However, the direct link between ITIH4 and decreased autophagy remained unexplained. Previous studies have suggested that circulating ITIH4 is capable of inhibiting mitogen-activated protein kinases (MAPK) signaling implicated in autophagy [50, 51]. Additionally, a previous study on mouse fibroblasts showed that YAP/TAZ knockdown significantly impairs autophagy, suggesting that the reduction of autophagy by ITIH4 could also be explained through its regulatory effects on various cellular signaling pathways, including the Hippo pathway as confirmed in our study [52]. Together, these findings suggested that ITIH4 might indirectly suppress autophagy through the regulation of the Hippo pathway, thereby improving lung injury.

There were some limitations in our study. First, further validation using human alveolar epithelial cells or organoid models would be beneficial to strengthen translational relevance. Second, the scRNA-Seq analysis, which provided an initial insight into cellular responses, might require further validation with additional in vitro replicates, larger sample sizes, and functional assays for confirmation.

## Conclusion

In conclusion, ITIH4 decreased acute lung damage and inflammatory responses in mice exposed to PM, particularly Fe, by involving the Hippo signaling pathway in AECII to resolve lung damage. Our findings suggested that understanding the mechanism of ITIH4 on gene and cellular responses, as well as the Hippo signaling pathway, may provide insights into treating acute lung injury by PM.

## Supplementary Information

Supplementary Material 1.

Supplementary Material 2.

## Data Availability

The datasets used and/or analyzed during the current study are available from the corresponding author on reasonable request.

## Abbreviations

AECI: Type I alveolar epithelial cells
AECII: Type II alveolar epithelial cells
ANOVA: Analysis of variance
As: Arsenic
BALF: Bronchoalveolar lavage
BSA: Bovine serum albumin
Cd: Cadmium
Co: Cobalt
COPD: Chronic obstructive pulmonary disease
Cr: Chromium
Cu: Copper
CXCL1: Chemokine (C-X-C motif) ligand 1
DEP: Diesel exhaust particle
ELISA: Enzyme-linked immunosorbent assay
Fe: Iron
FeCl_3_: Iron salt
FEV_1_: Forced expiratory volume in 1 s
FVC: Forced vital capacity
GO: Gene ontology
H&E: Hematoxylin and eosin
ICP-MS: Inductively coupled plasma mass spectrometry
IF: Immunofluorescence
IL: Interleukin
ITIH4: Inter-alpha-trypsin inhibitor heavy chain H4
KC: Keratinocyte chemoattractant
MAPK: Mitogen-activated protein kinases
Mn: Manganese
mWOB: Minute work of breathing
NADPH: Nicotinamide adenine dinucleotide phosphate
Ni: Nickel
Pb: Lead
PBMC: Peripheral blood mononuclear cells
PBS: Phosphate-buffered saline
PM: Particulate matter
PM_2.5_: Particulate matter with aerodynamic diameter of ≤ 2.5 μm
rITIH4: Recombinant inter-alpha-trypsin inhibitor heavy chain H4
sc: Single-cell
Se: Selenium
V: Vanadium
Zn: Zinc
