# Supplementary material for: ITIH4 attenuates acute lung injury by Fe-containing particulate matter in mice via Hippo pathway in type II alveolar epithelial cells
Source: Respir Res. 2025 May 28;26:201. doi: 10.1186/s12931-025-03256-z (PMC12121068; doi:10.1186/s12931-025-03256-z)
Supplement: Supplementary file 1 — Supplementary Material 1. [file 12931_2025_3256_MOESM1_ESM.docx]

**ITIH4 attenuates acute lung injury by Fe-containing particulate matter in mice via Hippo pathway in type II alveolar epithelial cells**

Vincent Laiman^1,2,#^ (MD, PhD), Syue-Wei Peng^3,#^ (PhD), Lina Choridah^1,2^ (MD, PhD), Didik Setyo Heriyanto^2,4^ (MD, PhD), Fara Silvia Yuliani^2,5^ (PhD), Kang-Yun Lee^6,7^ (MD, PhD), Ching-Huang Lai^8^ (PhD), Jer-Hwa Chang^3,9^ (MD, MSc), Yueh-Lun Lee^10^ (PhD), Shu-Chuan Ho^3,7^ (PhD), Sheng-Ming Wu^6,7^ (PhD), Chia-Li Han^11^ (PhD), Cheng-Wei Lin^12,13,14^ (PhD)*,* Kian Fan Chung^15^ (MD., DSc.), Hsiao-Chi Chuang^2,3,7,14,15*^ (PhD)

^1^Department of Radiology, Faculty of Medicine, Public Health, and Nursing, Universitas Gadjah Mada – Dr. Sardjito Hospital, Yogyakarta, Indonesia

^2^Collaboration Research Center for Precision Oncology based Omics - PKR PrOmics, Universitas Gadjah Mada, Yogyakarta, Indonesia

^3^School of Respiratory Therapy, College of Medicine, Taipei Medical University, Taipei, Taiwan

^4^Department of Anatomical Pathology, Faculty of Medicine, Public Health, and Nursing, Universitas Gadjah Mada – Dr. Sardjito Hospital, Yogyakarta, Indonesia

^5^Department of Pharmacology and Therapy, Faculty of Medicine, Public Health, and Nursing, Universitas Gadjah Mada, Yogyakarta, Indonesia

^6^Division of Pulmonary Medicine, Department of Internal Medicine, School of Medicine, College of Medicine, Taipei Medical University, Taipei, Taiwan

^7^Division of Pulmonary Medicine, Department of Internal Medicine, Shuang Ho Hospital, Taipei Medical University, New Taipei City, Taiwan

^8^School of Public Health, National Defense Medical Center, Taipei, Taiwan

^9^Division of Pulmonary Medicine, Departments of Internal Medicine, Wan Fang Hospital, Taipei Medical University, Taipei, Taiwan

^10^Department of Microbiology and Immunology, School of Medicine, College of Medicine, Taipei Medical University, Taipei, Taiwan

^11^Master Program in Clinical Genomics and Proteomics, College of Pharmacy, Taipei Medical University, Taipei, Taiwan

^12^Graduate Institute of Medical Sciences, College of Medicine, Taipei Medical University, Taipei, Taiwan.

^13^Department of Biochemistry and Molecular Cell Biology, Taipei Medical University, Taipei, Taiwan.

^14^Cell Physiology and Molecular Image Research Center, Wan Fang Hospital, Taipei Medical University, Taipei, Taiwan.

^15^National Heart and Lung Institute, Imperial College London, London, UK

**^#^These authors contributed equally to this work.**

***Corresponding Author**

*Hsiao-Chi Chuang, PhD*

Inhalation Toxicology Research Lab (ITRL), School of Respiratory Therapy, College of Medicine, Taipei Medical University, 250 Wuxing Street, Taipei 11031, Taiwan.

Telephone: +886-2-27361661 ext. 3512. Fax: +886-2-27391143. E-mail: [chuanghc@tmu.edu.tw](mailto:r92841005@ntu.edu.tw)

**Email of all authors**

Vincent Laiman ([vincentharun29@mail.ugm.ac.id](mailto:vincentharun29@mail.ugm.ac.id)); Syue-Wei Peng ([kiop995320@gmail.com](mailto:kiop995320@gmail.com)), Lina Choridah ([linachoridah@ugm.ac.id](mailto:linachoridah@ugm.ac.id)); Didik Setyo Heriyanto ([didik_setyoheriyanto@mail.ugm.ac.id](mailto:didik_setyoheriyanto@mail.ugm.ac.id)); Fara Silvia Yuliani ([fara.silvia.y@mail.ugm.ac.id](mailto:fara.silvia.y@mail.ugm.ac.id)); Kang-Yun Lee ([leekangyun@tmu.edu.tw](mailto:leekangyun@tmu.edu.tw)); Ching-Huang Lai ([lgh@mail.ndmctsgh.edu.tw](mailto:lgh@mail.ndmctsgh.edu.tw)); Jer-Hwa Chang ([m102094030@tmu.edu.tw](mailto:m102094030@tmu.edu.tw)); Yueh-Lun Lee ([yllee@tmu.edu.tw](https://hub.tmu.edu.tw/en/persons/yueh-lun-lee)); Shu-Chuan Ho ([shu-chuan@tmu.edu.tw](mailto:shu-chuan@tmu.edu.tw)); Sheng-Ming Wu ([15711@s.tmu.edu.tw](mailto:15711@s.tmu.edu.tw)); Chia-Li Han ([was@tmu.edu.tw](https://hub.tmu.edu.tw/en/persons/chia-li-han)); Cheng-Wei Lin ([cwlin@tmu.edu.tw](https://hub.tmu.edu.tw/en/persons/cheng-wei-lin)); Kian Fan Chung ([f.chung@imperial.ac.uk](mailto:f.chung@imperial.ac.uk)); Hsiao-Chi Chuang ([chuanghc@tmu.edu.tw](mailto:chuanghc@tmu.edu.tw))

**Materials and methods**

*S1. Lung function examination*

The pulmonary function was measured by Flexivent (SCIREQ; Sterling, VA, US). Calibration was performed before the experiment according to the manufacturer’s manual. The mice were anesthetized with 50 μg/kg Zoletil (Zoletil 100, Virbac Sante Animale, France) and 50 μg/kg Rompun (Rompun®, Bayer, Korea, Korea) by intraperitoneal administration. A mouse was held at rest until the righting reflex was lost and then was placed to experimental chamber. The mice were then inserted with the 24-gauge soft catheter through the trachea and connected with the ventilator in Flexivent.

*S2. Bronchoalveolar lavage (BALF) differential cell count*

The BALF was obtained from the mice by administration of 1 mL of PBS followed with 3 times of gentle lavage. The collected BALF samples were then centrifuged at 1500 rpm for 10 min at 4°C. The supernatant was collected and kept in -80ºC for biochemical analyses, whereas the pellet was suspended in PBS for cell counting. BALF cell counting was conducted using a hematology analysis (ProCyte Dx; IDEXX Laboratories; Westbrook, Maine, USA).

*S3. Serum and peripheral blood mononuclear (PBMC) collection*

The submandibular method was used for blood collection. The collected blood samples were stored at room temperature for 1 hr before following centrifugation at 1500 rpm at room temperature for 10 min. The serum of the blood was then collected and kept in -80ºC for further analyses. For PBMC collection, the collected blood samples were added to tube containing Dulbecco's Modified Eagle Medium (DMEM) medium and heparin of 30 USP/mL of blood. The samples were then slowly added to tube containing Ficoll (Ficoll® Paque Plus, Sigma, St. Louis, MO, USA) without breaking its surface integrity. Density-gradient centrifugation with 2000 rpm for 20 mins were then performed on the samples, which relies on physical characteristics such as size and density to sort cell populations. The buffy coat layer containing the PBMC was then obtained and fixed with 4% paraformaldehyde.

*S4. Metals in PBMC by bulk inductively coupled plasma mass spectrometry (ICP-MS)*

The obtained PBMC was used for analysis of metal contents by ICP-MS using iCAP RQ ICP-MS spectrometer (Thermo Fisher Scientific, MA, USA). The samples were prepared by diluting it in tenfold (1 mL specimen + 9 mL PBS) and added to pre-cleaned polypropylene tubes (Nalge Nunc International, Rochester, NY, USA) before being thermally processed by high-pressure microwave to assist digestion. Deionized water blanks and a certified ICP quality control standard (QCS-01-1, AccuStandard, CT, USA) were respectively used to detect contamination and the accuracy of the analyses. The tubing and nebulizer were cleaned by pumping through 10 mL 3.5% nitric acid (HNO_3_). After igniting the ICP, the system was preconditioned by aspirating the diluted HNO_3_ for approximately 20 min. After performing mass calibration, the sequence was acquired, starting with a set of synthetic blanks, standards, followed by reference materials and the samples. The ICP-MS was used to determine twelve metal concentrations: As, Cd, Co, Cr, Cu, Fe, Mn, Ni, Pb, Se, V, Zn. The relative percentage difference was <10%.

*S5. Single-cell Fe in PBMC by single-cell (sc)ICP-MS*

The PBMC sample was used for identification of single-cell Fe content using scICP-MS. In principle, the cells are embedded in droplets generated by conventional nebulization and introduced into the plasma. After vaporization, each cell generates an ion cloud which is detected as an individual spike signal using short dwell times, with the intensity being proportional to the quantity of the analyte ions present in a single-cell, and the number of spike signals is proportional to the number of cells containing the analyte of interest. The samples were prepared in the same way as those prepared in ICP-MS. Briefly, samples were diluted tenfold (1 mL specimen + 9 mL PBS) and added to pre-cleaned polypropylene tubes (Nalge Nunc International, Rochester, NY, USA) before being thermally processed by high-pressure microwave to assist digestion. Deionized water blanks and 56Fe (12310-7439-89-6, Sigma, St. Louis, MO, USA) was used as standard for Fe content in cell determination. Before starting, the tubing and nebulizer were cleaned by pumping through 10 mL 3.5% nitric acid (HNO_3_). After igniting the ICP, the system was preconditioned by aspirating the diluted HNO_3_ for approximately 20 min. After performing mass calibration, the sequence was acquired, starting with a set of synthetic blanks, standards, followed by the samples.

*S6. Single-cell RNA sequencing (scRNA-Seq)*

Lung samples were taken from mice after the lung was perfused to eliminate red blood cells. The lung was dissociated using lung dissociation kit (130-095-927, Miltenyi, MD, USA), then filtered through a 70 µm cell strainer. Cell pellets were then resuspended in red blood cell lysis solution, washed, and enriched with CD326 EpCAM MicroBeads (130-105-958, Miltenyi, MD, USA), followed by the removal of dead cells (130-090-101, Miltenyi, MD, USA). The cellular viability of the samples, determined by the trypan blue staining was more than 90%. Cells were analyzed using 10x Single Cell 3’ v3 sequencing kit following the protocol provided by the company. Library quality was assessed following cDNA synthesis and after completion of the 3’ Gene Expression Libraries on the Agilent TapeStation system employing a DNA High Sensitivity D5000 and D1000 chips, respectively (all Agilent). Single cell libraries were sequenced on Illumina NovaSeq 6000 instrument using read 1: 28bp and read 2: 90bp length sequencing and aiming at 20,000 reads/cell. To analyse the scRNA-Seq information, the Cell Ranger software pipeline (version 2.0) provided by 10X Genomics was employed. Briefly, the data was de-barcoded, and the nucleotide reads were mapped to the genome and transcriptome with the Spliced Transcripts Alignment to a Reference (STAR) aligner software (CSC, Espoo, Finland). The R package Seurat (version 1.4.0.14; https://cran.r-project.org/web/ packages/Seurat/index.html)[1] was used for processing the UMI count matrix and removal of potential multiple captures. Normalized aggregate data across samples was generated to produce a matrix of gene counts versus cells. The following quality control criteria were applied to cell prefiltering, including 500–4000 expressed genes, less than 20% of UMIs mapped to mitochondrial genes, and less than 20,000 total UMIs. Automatic annotation on cell types of clusters from single-cell RNA sequencing data was performed using scCATCH [2]. Loupe™ Cell Browser v2.1 (10X Genomics) was used to perform analysis in gene expressions in AECII *Epcam* positive (*Epcam*^+^) and *Sftpc* positive (*Sftpc*^+^) cells.

*S7. Western blotting*

Protein concentrations of the lung lysates were determined using the BCA Protein Assay Reagent Kit (Bio-Rad, Hercules, CA, USA). Protein extracts were analyzed on sodium dodecyl-sulfate (SDS)-polyacrylamide gels and transferred to PolyScreen series polyvinylidene difluoride (PVDF) blotting membranes (PerkinElmer, Waltham, MA, USA). Blocking of the blots were performed with 5% skim milk in tris-buffered saline with 0.1% Tween® 20 (TBST) at room temperature for 1 hr followed by incubation with the primary antibody diluted in TBST overnight at 4°C. Primary antibodies used were anti-E-cadherin (AB76319, Abcam, Cambridge, UK), ⍺-catenin (#3236, Cell Signaling, MA, USA), YAP (66900-1-Ig, Proteintech, IL, USA), pYAP (AB76252, Abcam, Cambridge, UK), TAZ (#83669, Cell Signaling, MA, USA), pTAZ (#59971, Cell Signaling, MA, USA), Transferrin (GTX101035, Genetex, CA, USA), CD71 (GTX102596, Genetex, CA, USA), LC3B (3868S, Cell signaling, MA, USA), ITIH4 (sc-515060, Santa Cruz Biotechnology, TX, USA), and β-actin (60008-1-lg, Proteintech, IL, USA). The blots were washed 5× with TBST for 5 min and incubated with HRP-labelled secondary antibody AffiniPure anti-mouse IgG (115-005-003, Jackson ImmunoResearch) and AffiniPure anti-rabbit IgG (111-035-003, Jackson ImmunoResearch, West Grove, PA, USA) diluted in TBST for 1 hr at room temperature. Immunoreactivity was observed through enhanced chemiluminescence (ECL). Images were taken with the ChemiDoc™ MP Imaging System (Bio-Rad, CA, USA). Quantitative data were obtained using Image-Pro vers. 4 (Media Cybernetics, Inc., MD, USA) for Windows. All data were adjusted to the control (multiples of change of the control).

*S8. Lung damage assessment*

Lung sections were fixed using 10% buffered formalin by tracheal instillation at a pressure of 25 cmH_2_O for 10 min. Following that, lung tissues were embedded in paraffin, sectioned, and stained with hematoxylin and eosin (H&E). Lung H&E images were obtained by Motic Easyscan Pro and Motic DSAssistant software (Motic, Xiamen, Fujian, China). Lung damage assessment was performed by utilizing K-means clustering algorithm using ImageJ software (National Institute of Health, Bethesda, MD, USA) to rank and sort points on H&E slides according to staining intensity and density which varies with injury severity [3, 4]. All pixels from the tissue images were sorted into four clusters, comprising of a background component and three clusters that highly correlate with injury zones. An image of a single lobe is defined as a means of comparison and each image is clustered together with that image to avoid possibility of bias in the relative clustering of heterogeneously injured tissue.

*S9. Immunofluorescence (IF) staining*

For lung sections of B6.*Sftpc-CreER^T2^;Ai14(RCL-tdT)-D* mice, lungs were perfused with 4% paraformaldehyde for 4 hr, submerged in 30% sucrose solution overnight, and then embedded in optimal cutting temperature compound (Leica, Deer Park, IL, USA) before being sectioned using cryostat (CM3050S, Leica, Deer Park, IL, USA). Sections were then washed in PBS followed by blocking step using a 5% bovine serum albumin (BSA) solution. The blocking step was performed using a 5% BSA solution. Following that, the lungs were incubated with primary and secondary antibody. Primary antibodies used were anti-E-cadherin (AB76319, Abcam, Cambridge, UK), ⍺-catenin (#3236, Cell Signaling, MA, USA), YAP (66900-1-Ig, Proteintech, IL, USA), pYAP (AB76252, Abcam, Cambridge, UK), TAZ (#83669, Cell Signaling, MA, USA), and pTAZ (#59971, Cell Signaling, MA, USA). Fluorescein isothiocyanate (FITC)-conjugated species-specific secondary antibodies (AB150077 and AB150079, Abcam, Cambridge, UK), and 4’,6-diamidino-2-phenylindole (DAPI) (AB104139, Abcam, Cambridge, UK) for nuclear staining were used after primary antibody. Fluorescent images were captured at 20x magnification using a fluorescence microscope (Echo Revolve, Echo, San Diego, CA, USA).

References

1. Stuart T, Butler A, Hoffman P, Hafemeister C, Papalexi E, Mauck WM, Hao Y, Stoeckius M, Smibert P, Satija R: **Comprehensive Integration of Single-Cell Data.** *Cell* 2019, **177:**1888-1902.e1821.

2. Shao X, Liao J, Lu X, Xue R, Ai N, Fan X: **scCATCH: Automatic Annotation on Cell Types of Clusters from Single-Cell RNA Sequencing Data.** *iScience* 2020, **23**.

3. Liberti DC, Kremp MM, Liberti WA, Penkala IJ, Li S, Zhou S, Morrisey EE: **Alveolar epithelial cell fate is maintained in a spatially restricted manner to promote lung regeneration after acute injury.** *Cell Reports* 2021, **35**.

4. Chen L, Shan W, Liu P: **Identification of concrete aggregates using K-means clustering and level set method.** *Structures* 2021, **34:**2069-2076.
