# Supplementary material for: ITIH4 attenuates acute lung injury by Fe-containing particulate matter in mice via Hippo pathway in type II alveolar epithelial cells
Source: Respir Res. 2025 May 28;26:201. doi: 10.1186/s12931-025-03256-z (PMC12121068; doi:10.1186/s12931-025-03256-z)

E-cadherin
135 kDA

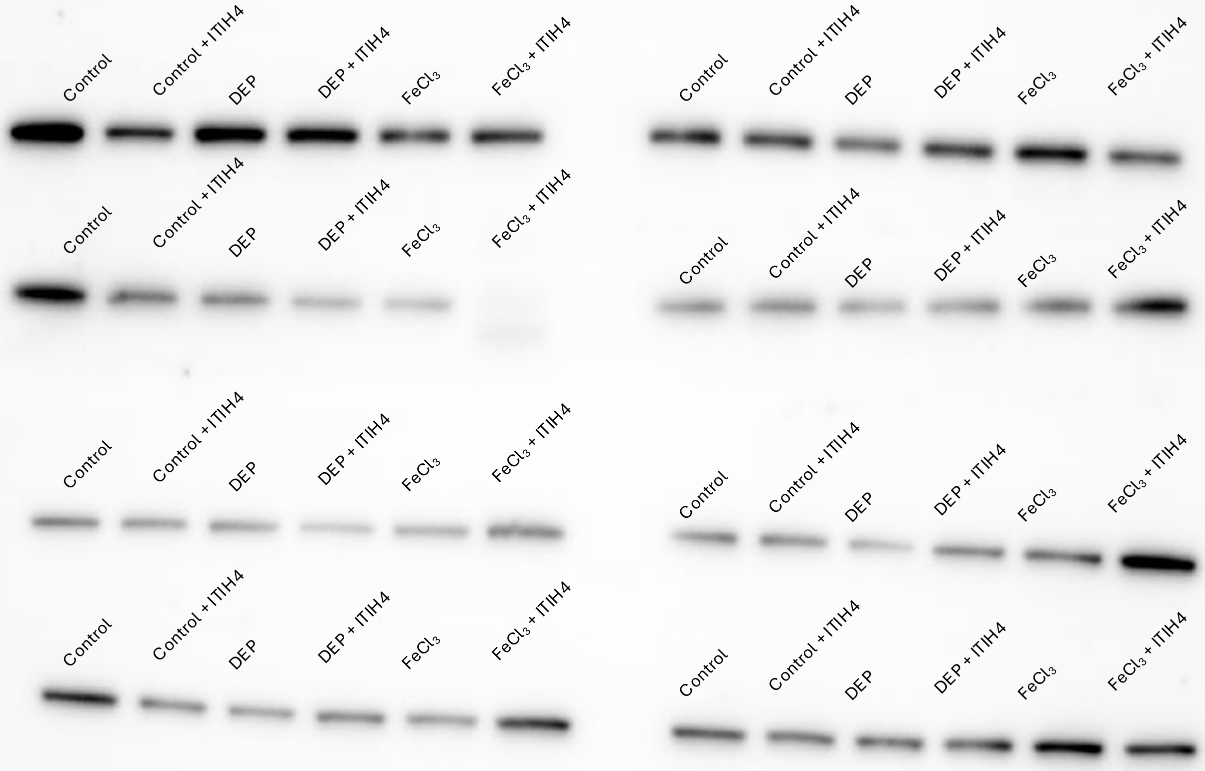

E-cadherin
135 kDA

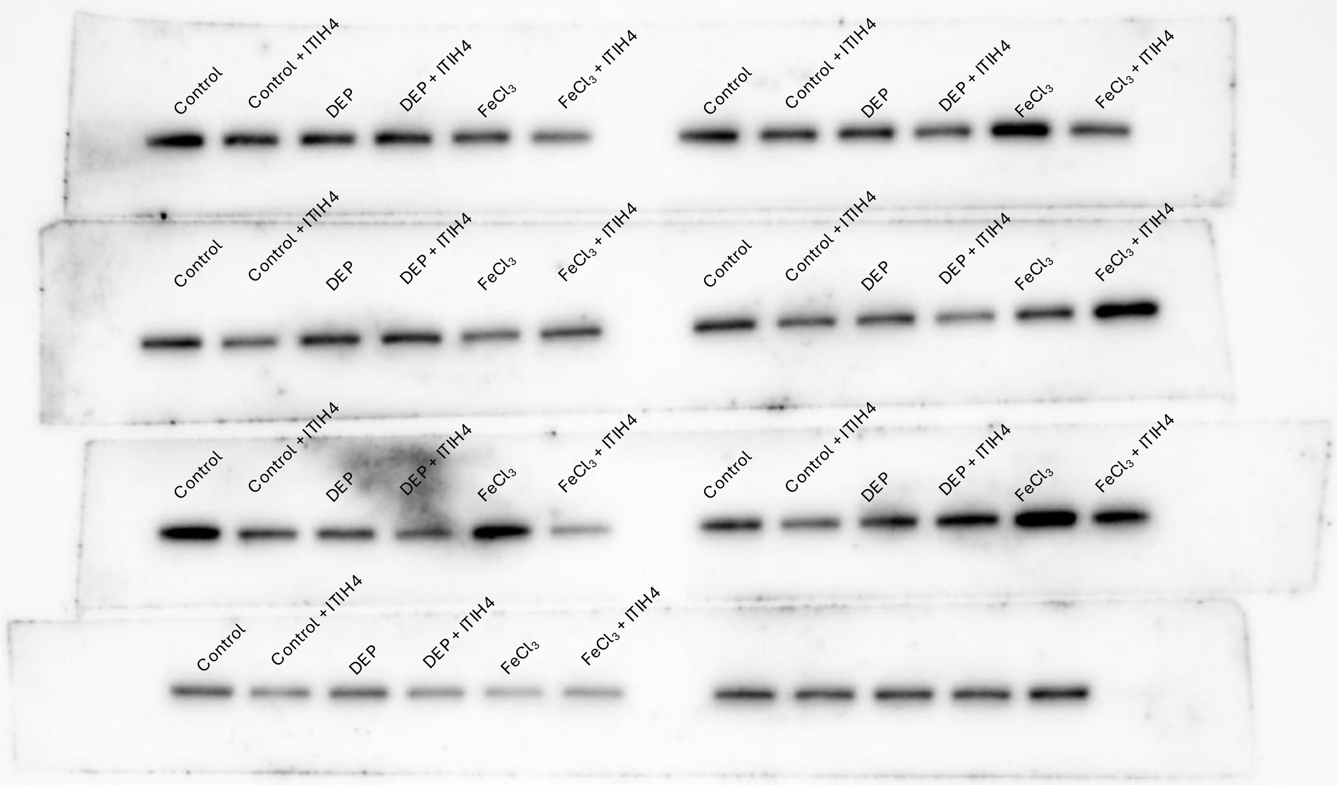

α-catenin
100 kDA

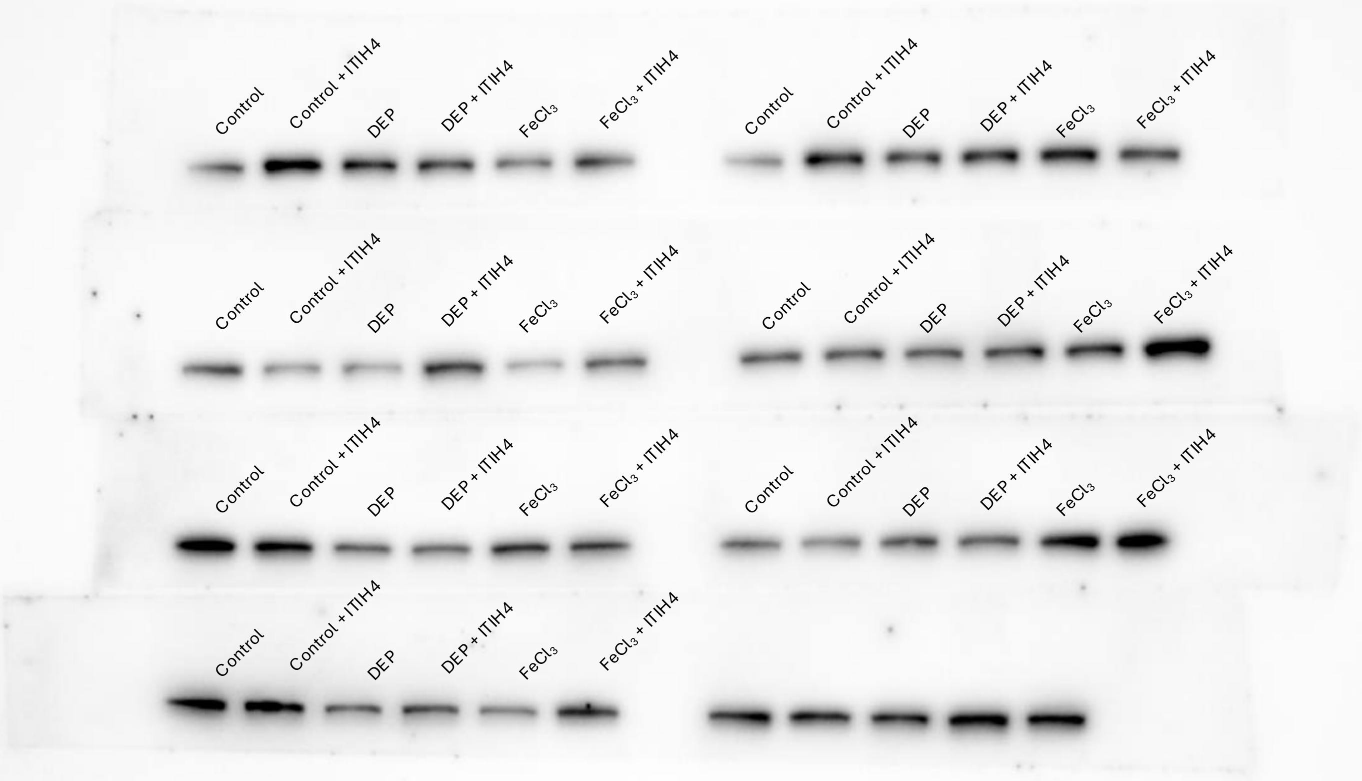

α-catenin
100 kDA

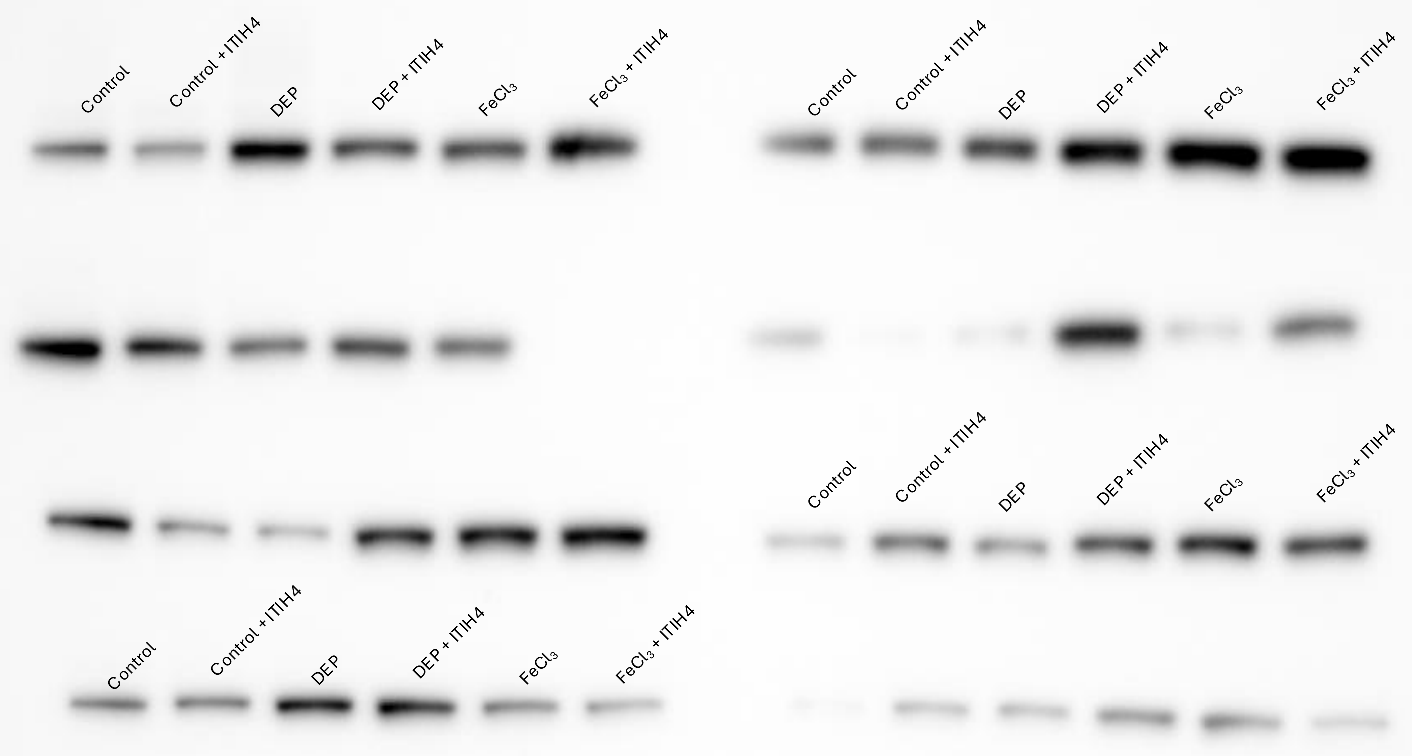

YAP 70kDA

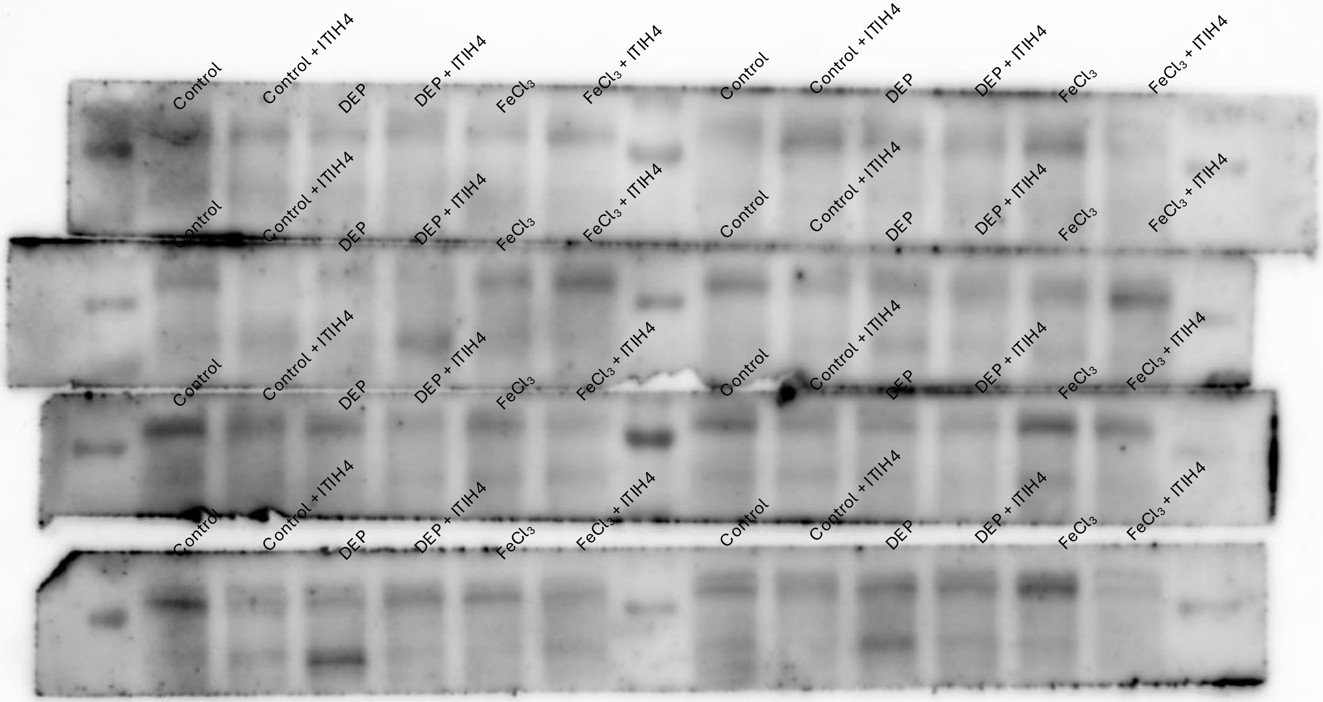
Control
Control + ITIH4
DEP
DEP + ITIH4
FeCl3
FeCl3 + ITIH4
Control
Control + ITIH4
DEP
DEP + ITIH4
FeCl3
FeCl3 + ITIH4
Control
Control + ITIH4
DEP
DEP + ITIH4
FeCl3
FeCl3 + ITIH4
Control
Control + ITIH4
DEP
DEP + ITIH4
FeCl3
FeCl3 + ITIH4
Control
Control + ITIH4
DEP
DEP + ITIH4
FeCl3
FeCl3 + ITIH4
Control
Control + ITIH4
DEP
DEP + ITIH4
FeCl3
FeCl3 + ITIH4
Control
Control + ITIH4
DEP
DEP + ITIH4
FeCl3
FeCl3 + ITIH4
Control
Control + ITIH4
DEP
DEP + ITIH4
FeCl3
FeCl3 + ITIH4

YAP 70kDA

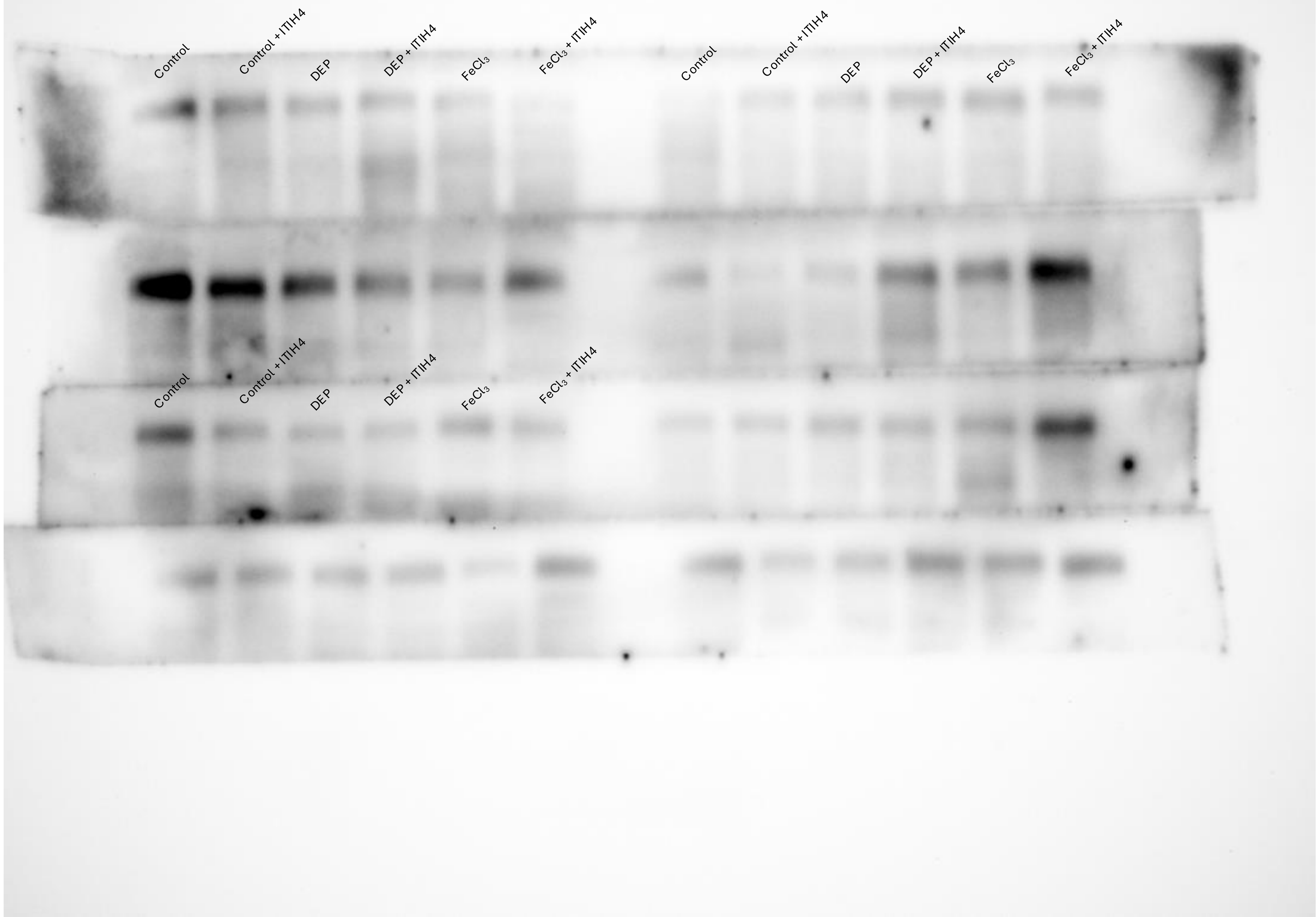
Control
Control +ITIH4
DEP
DEP + ITIH4
FeCl3
FeCl3 + ITIH4
Control
Control +ITIH4
DEP
DEP + ITIH4
FeCl3
FeCl3 + ITIH4
Control
Control +ITIH4
DEP
DEP + ITIH4
FeCl3
FeCl3 + ITIH4

pYAP 65kDA

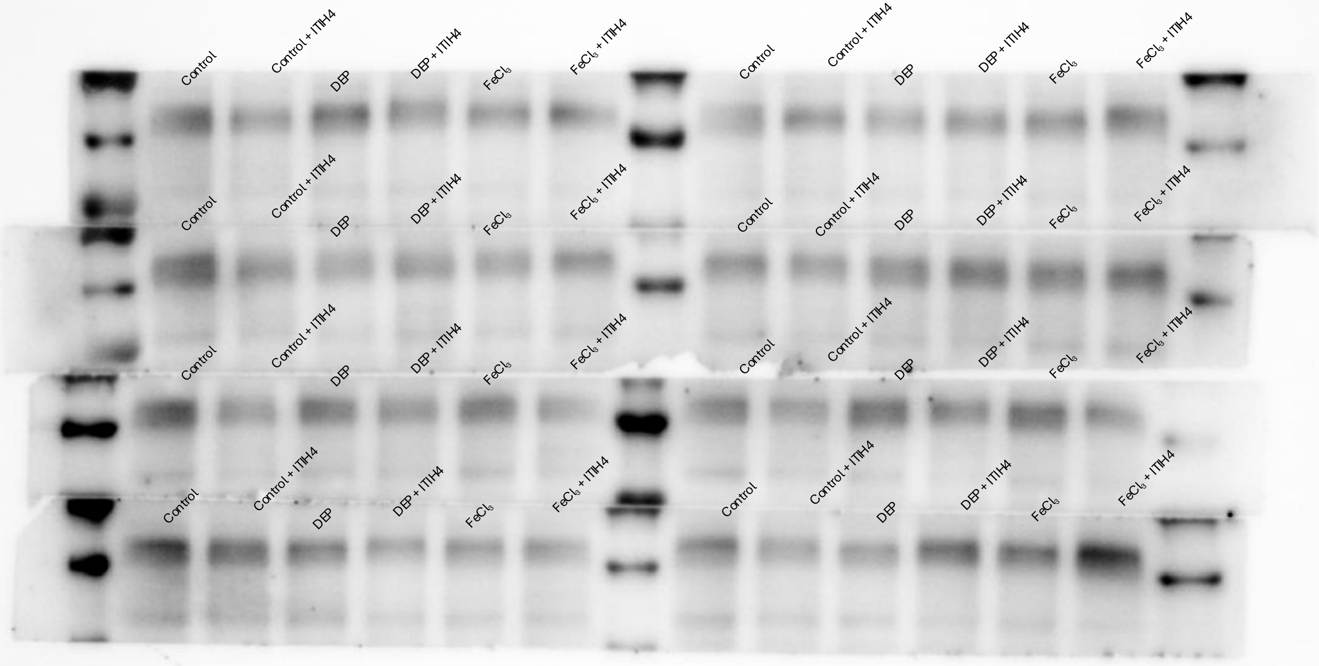

pYAP 65kDA

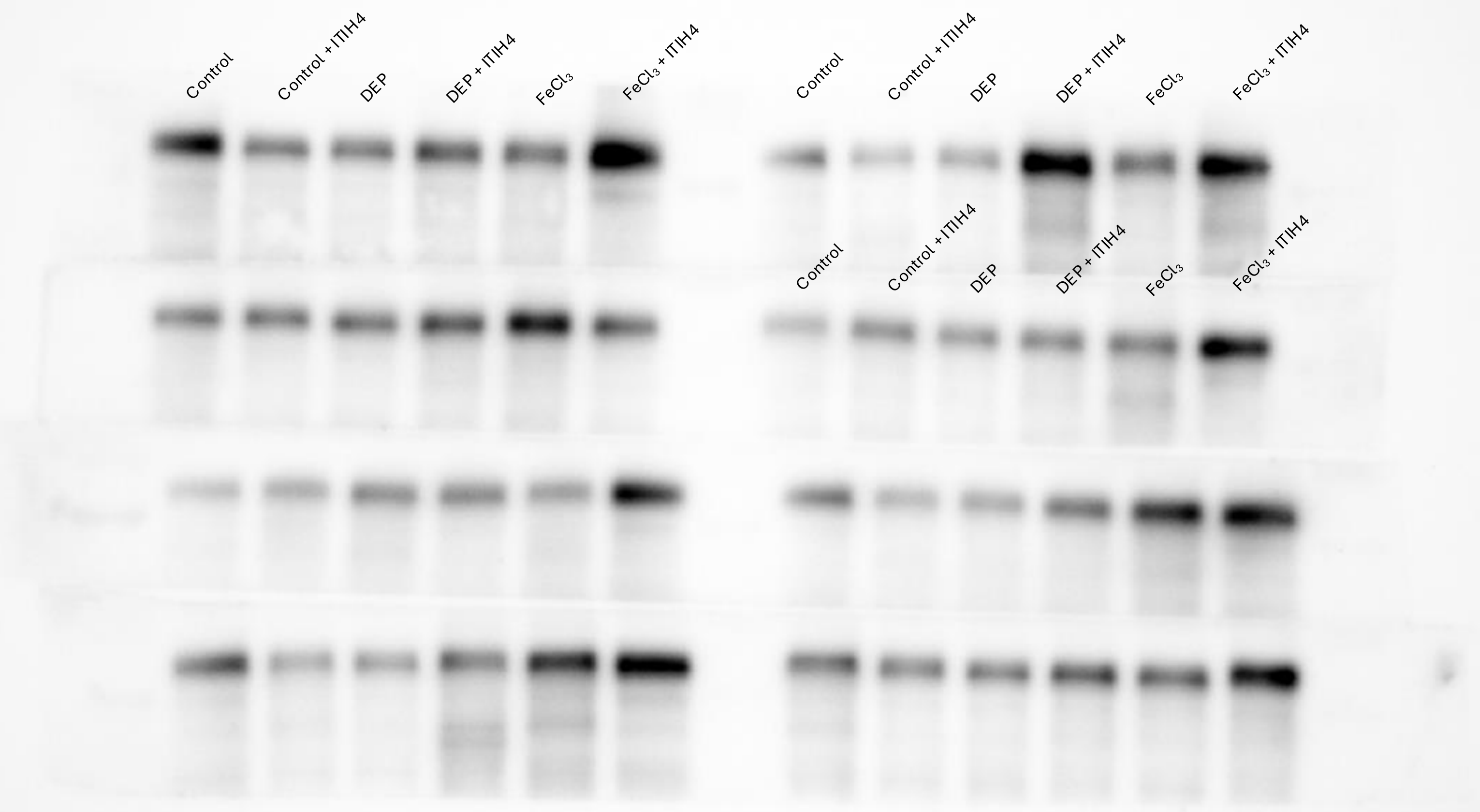

Control
Control + ITIH4
DEP
DEP + ITIH4
$FeCl_3$
$FeCl_3$ + ITIH4
Control
Control + ITIH4
DEP
DEP + ITIH4
$FeCl_3$
$FeCl_3$ + ITIH4
Control
Control + ITIH4
DEP
DEP + ITIH4
$FeCl_3$
$FeCl_3$ + ITIH4

TAZ 55 kDA

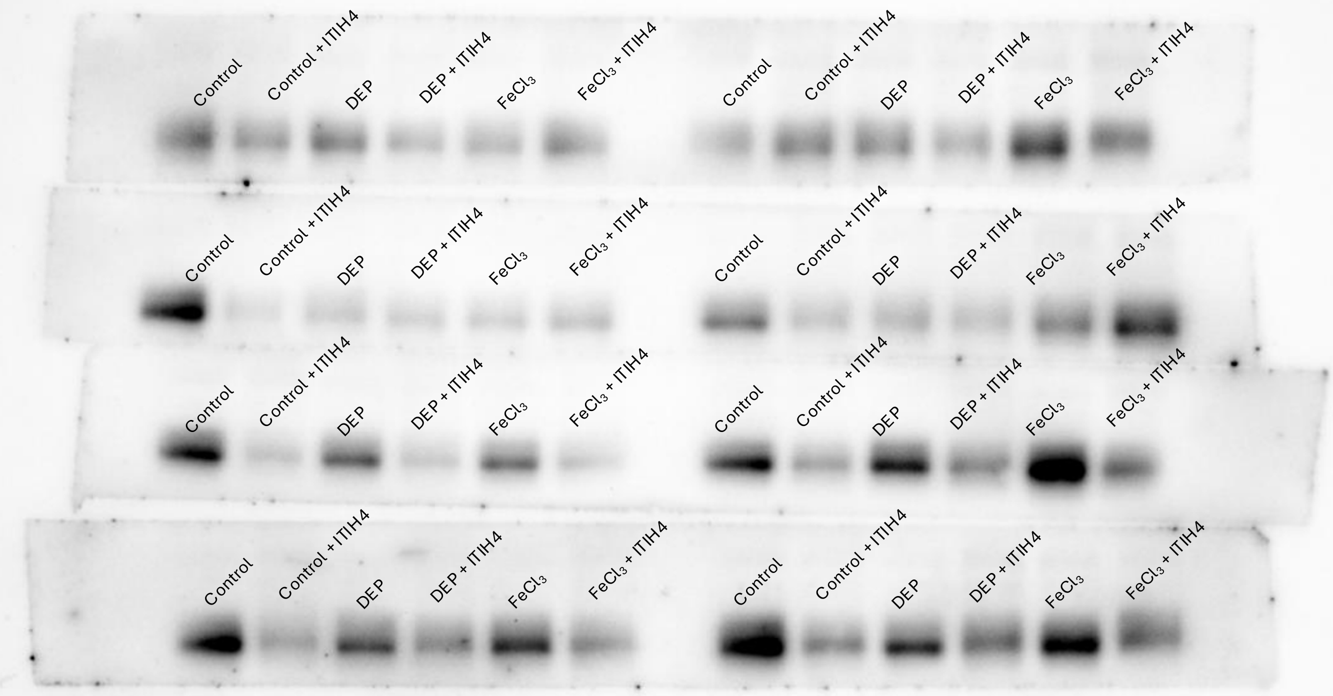

Control
Control + ITIH4
DEP
DEP + ITIH4
FeCl3
FeCl3 + ITIH4
Control
Control + ITIH4
DEP
DEP + ITIH4
FeCl3
FeCl3 + ITIH4

TAZ 55 kDA

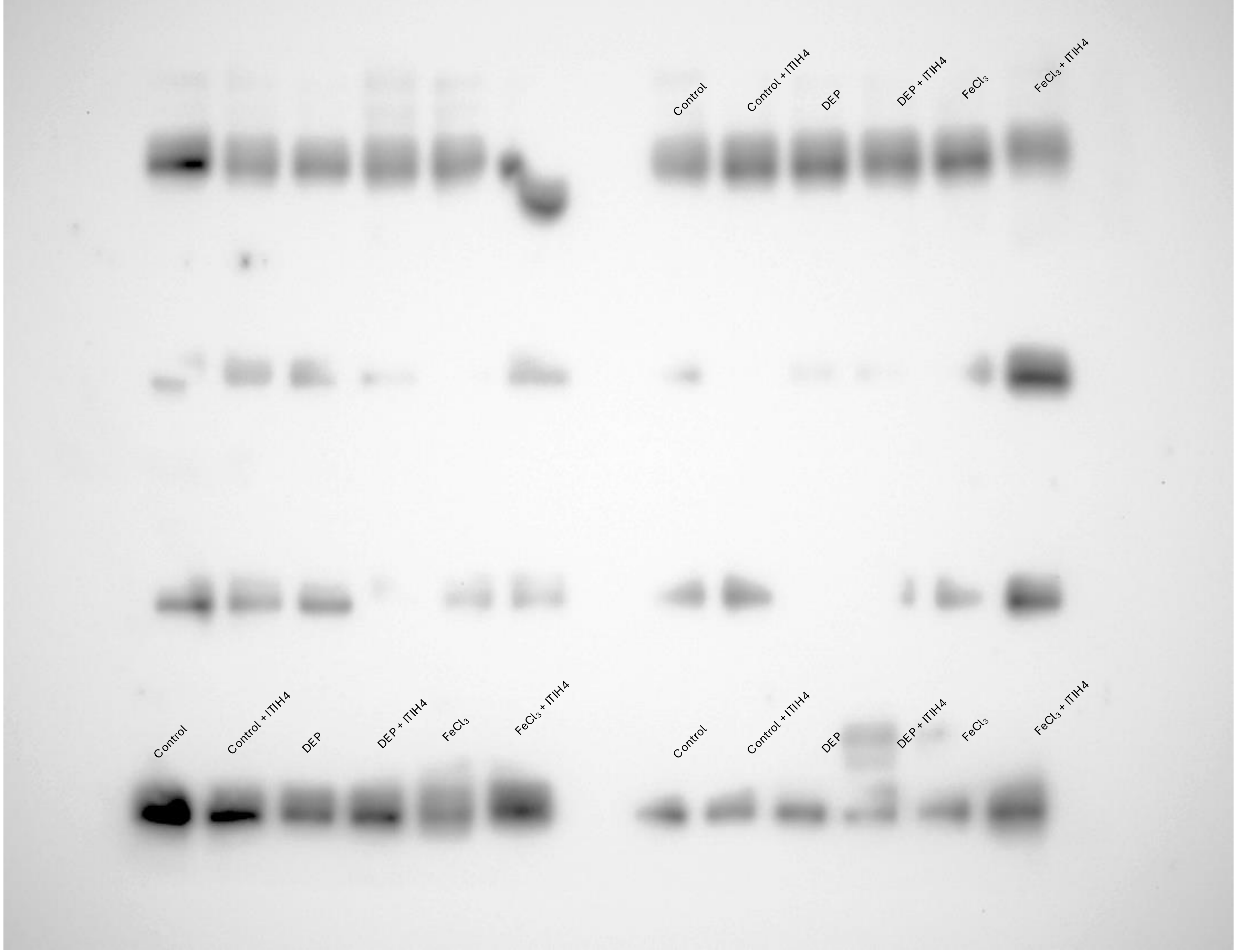
Control
Control + ITIH4
DEP
DEP + ITIH4
$FeCl_3$
$FeCl_3$ + ITIH4
Control
Control + ITIH4
DEP
DEP + ITIH4
$FeCl_3$
$FeCl_3$ + ITIH4
Control
Control + ITIH4
DEP
DEP + ITIH4
$FeCl_3$
$FeCl_3$ + ITIH4

pTAZ

65kDA

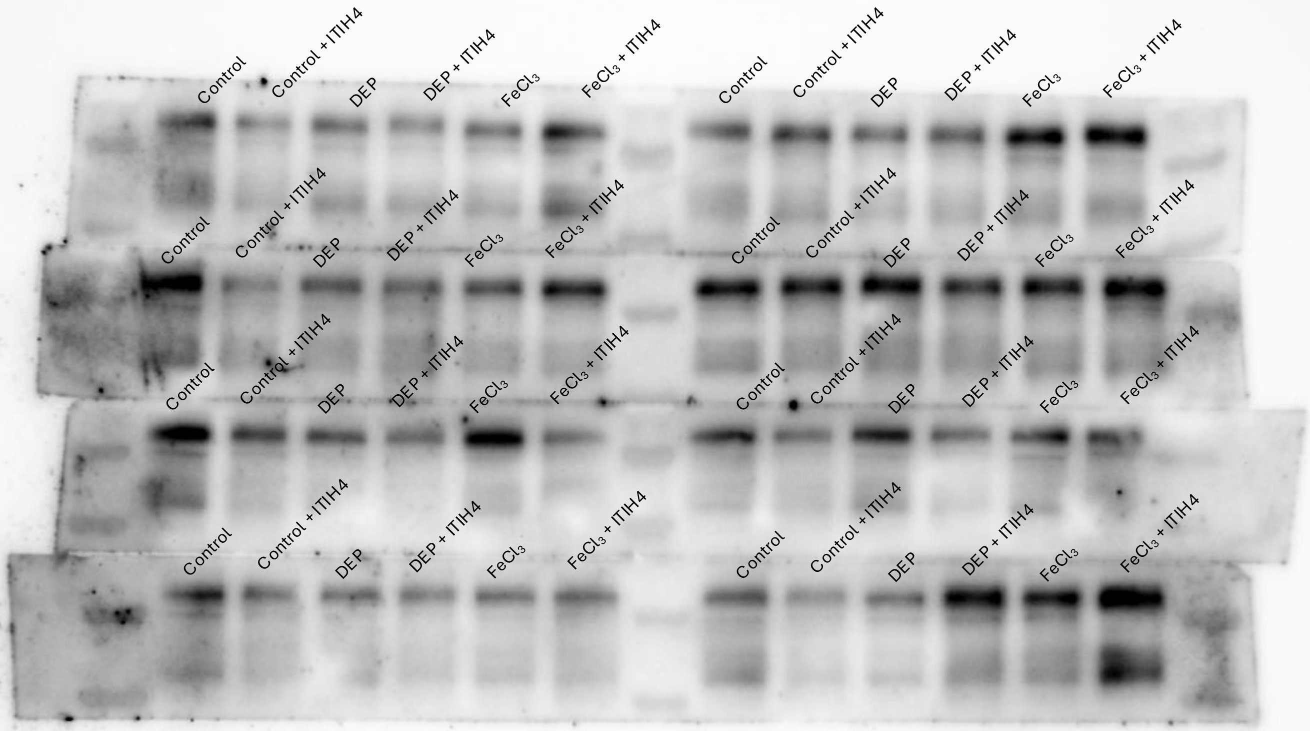

Control
Control + ITIH4
DEP
DEP + ITIH4
FeCl3
FeCl3 + ITIH4
Control
Control + ITIH4
DEP
DEP + ITIH4
FeCl3
FeCl3 + ITIH4
Control
Control + ITIH4
DEP
DEP + ITIH4
FeCl3
FeCl3 + ITIH4
Control
Control + ITIH4
DEP
DEP + ITIH4
FeCl3
FeCl3 + ITIH4
Control
Control + ITIH4
DEP
DEP + ITIH4
FeCl3
FeCl3 + ITIH4
Control
Control + ITIH4
DEP
DEP + ITIH4
FeCl3
FeCl3 + ITIH4
Control
Control + ITIH4
DEP
DEP + ITIH4
FeCl3
FeCl3 + ITIH4
Control
Control + ITIH4
DEP
DEP + ITIH4
FeCl3
FeCl3 + ITIH4

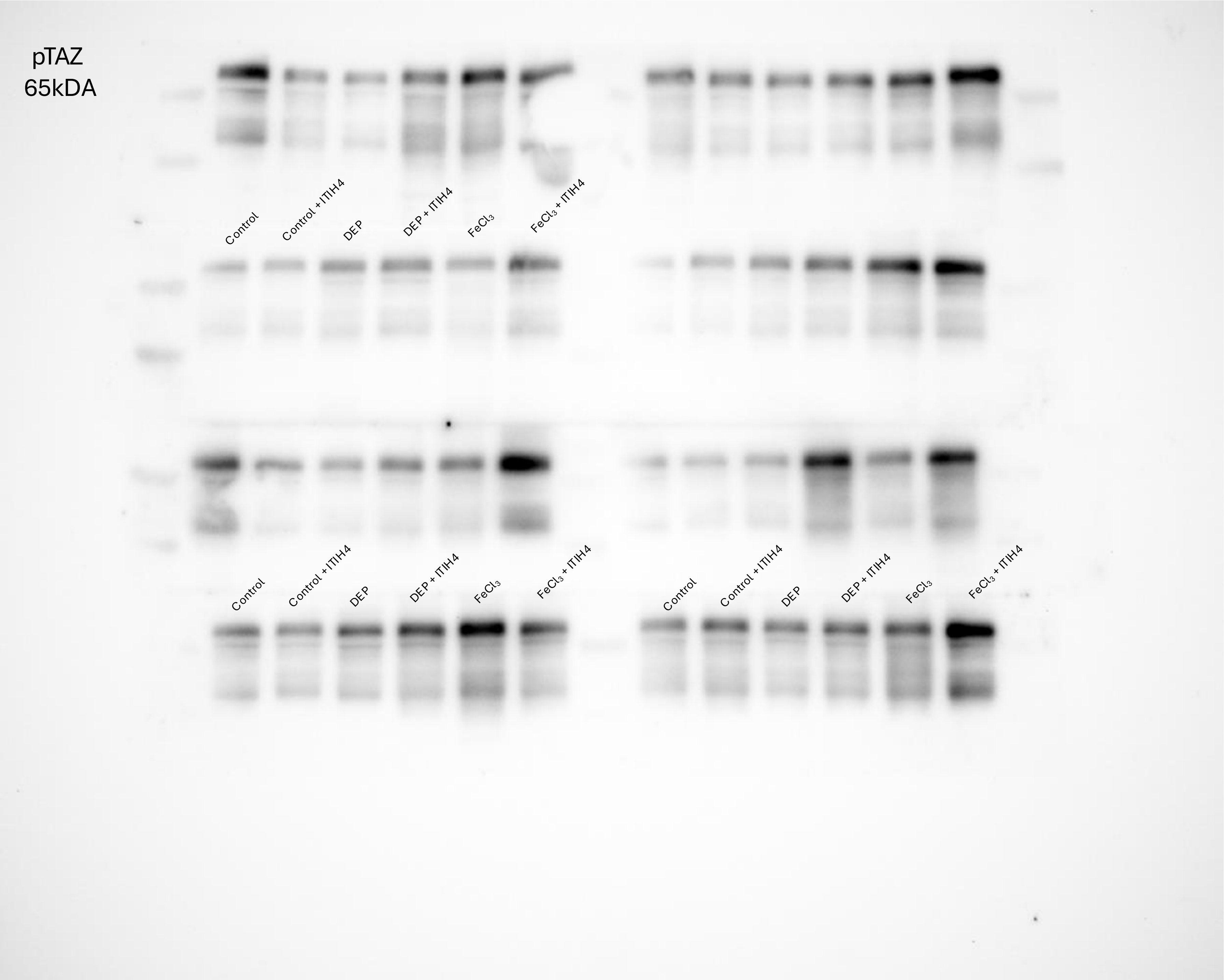
pTAZ
65kDA
Control
Control + ITIH4
DEP
DEP + ITIH4
$FeCl_3$
$FeCl_3$ + ITIH4
Control
Control + ITIH4
DEP
DEP + ITIH4
$FeCl_3$
$FeCl_3$ + ITIH4
Control
Control + ITIH4
DEP
DEP + ITIH4
$FeCl_3$
$FeCl_3$ + ITIH4

Transferrin
77kDA

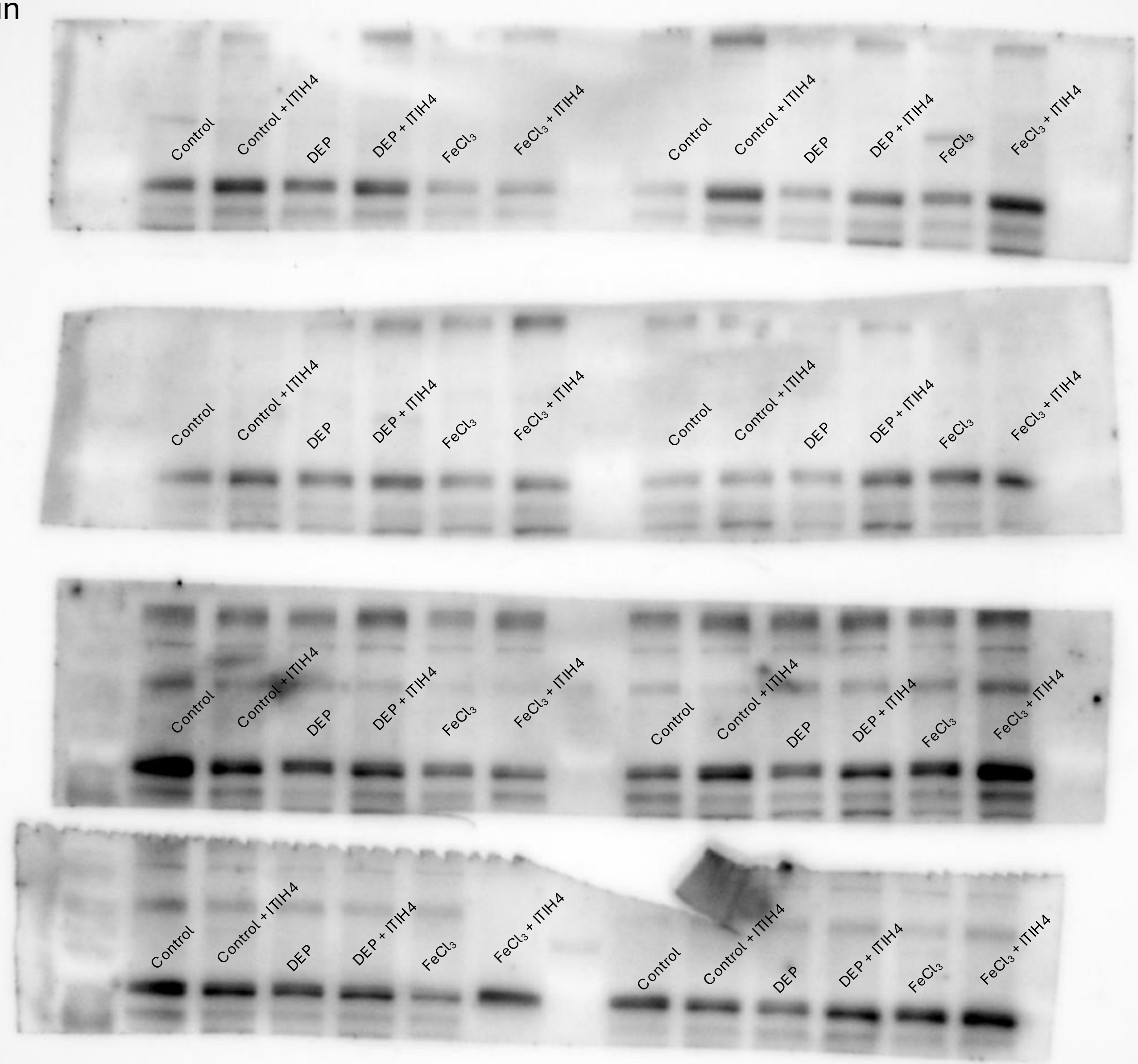

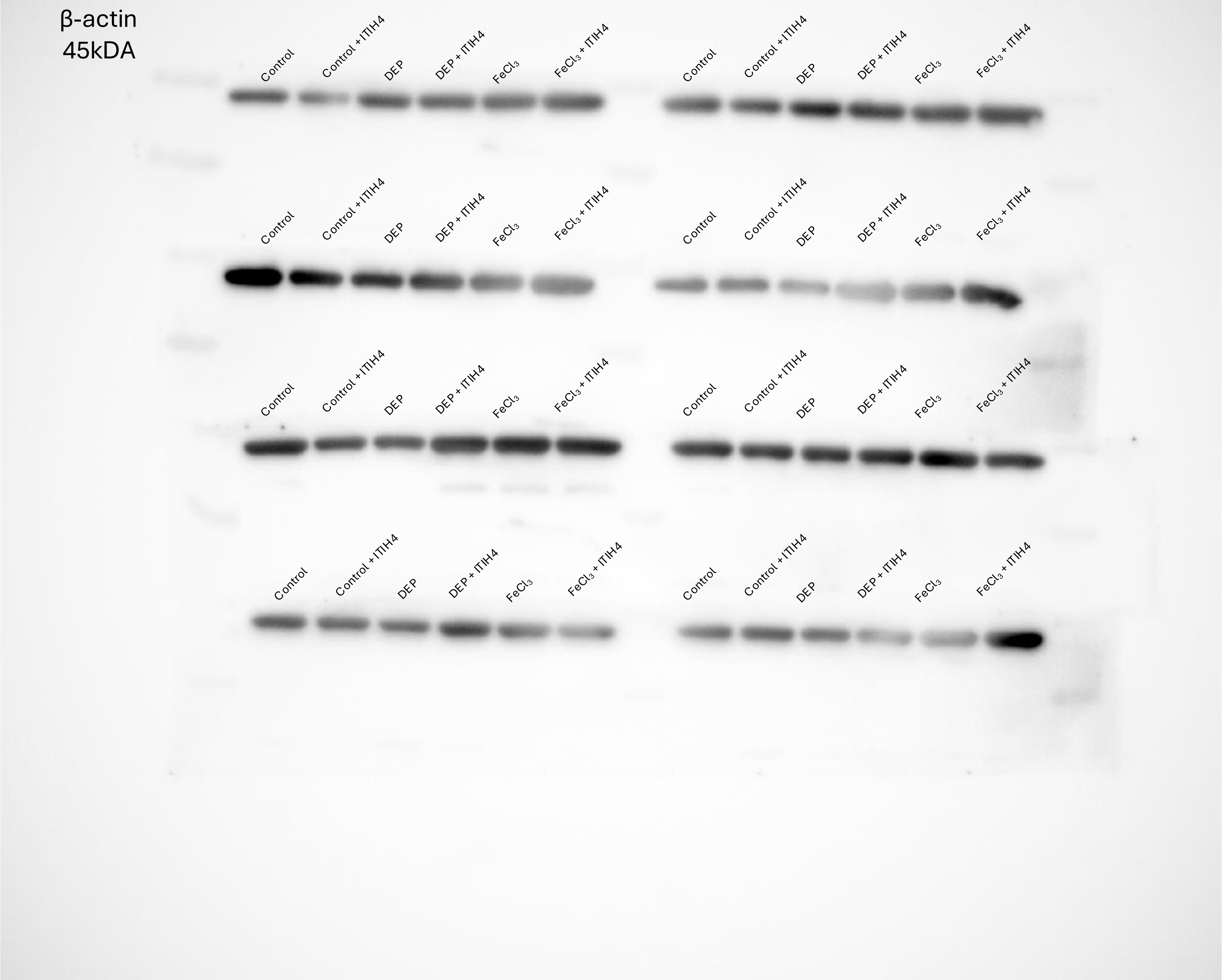

β-actin
45kDA
Control
Control + ITIH4
DEP
DEP + ITIH4
$FeCl_3$
$FeCl_3$ + ITIH4

Transferrin receptor
85 kDA

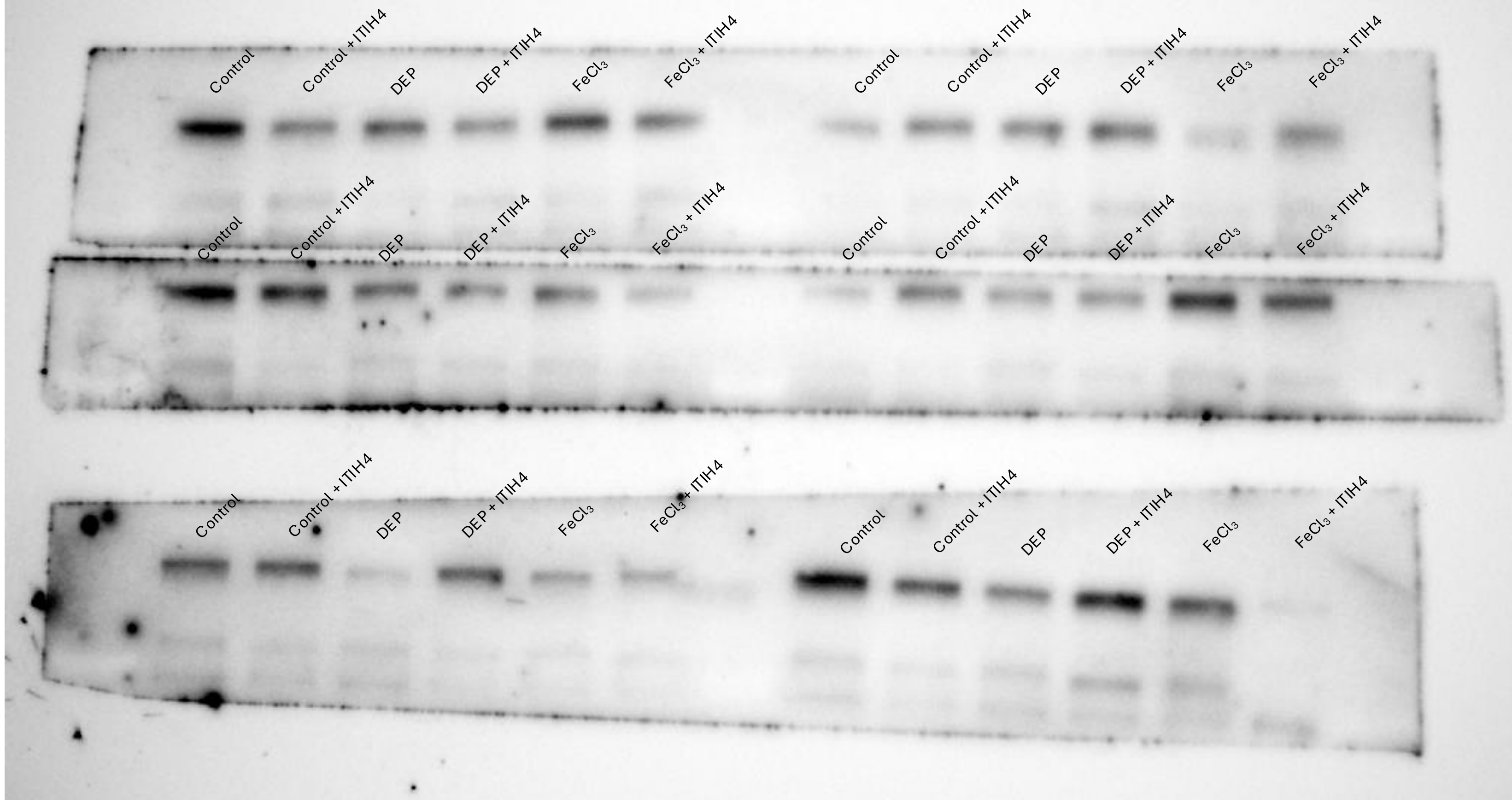

Transferrin receptor
85 kDA

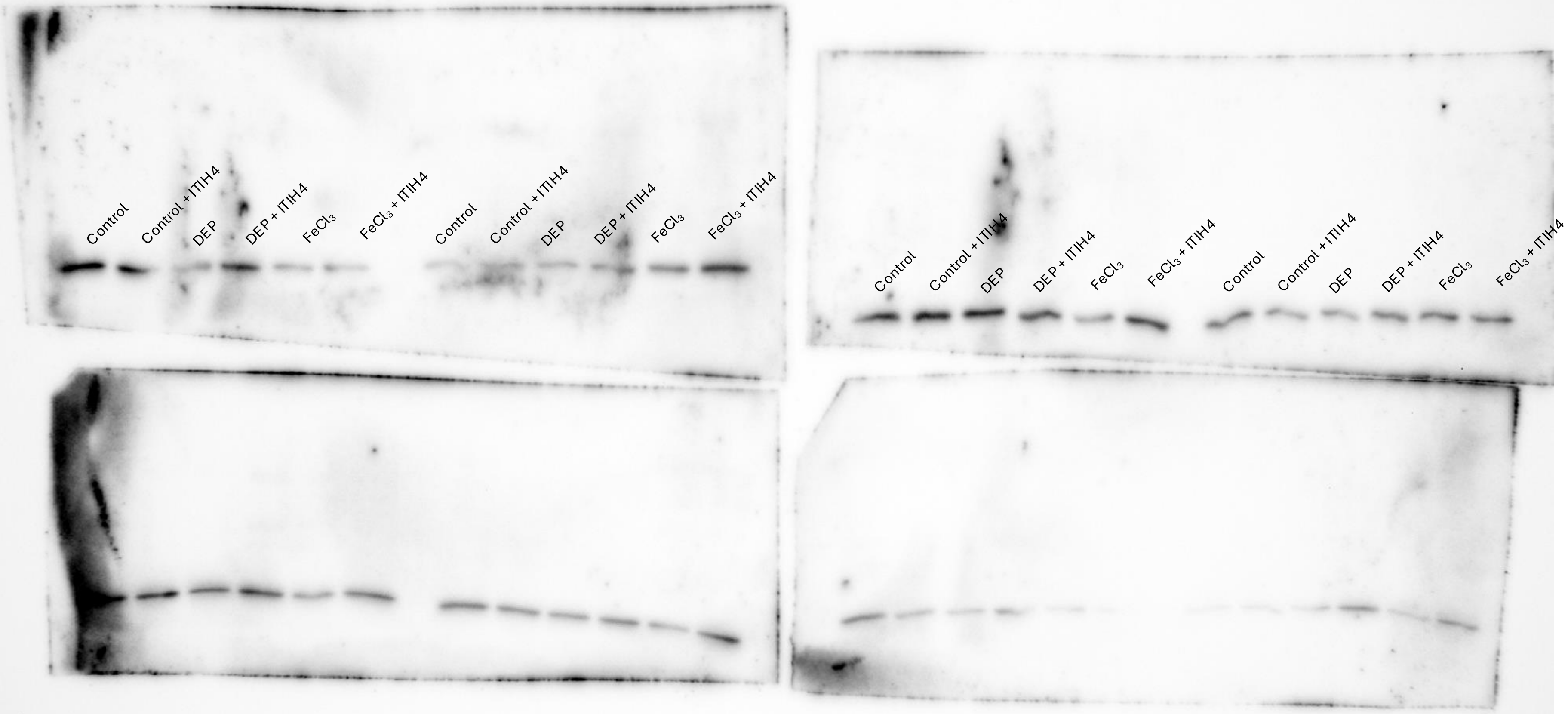

LC3B I 16kDA
LC3B II 14 kDA

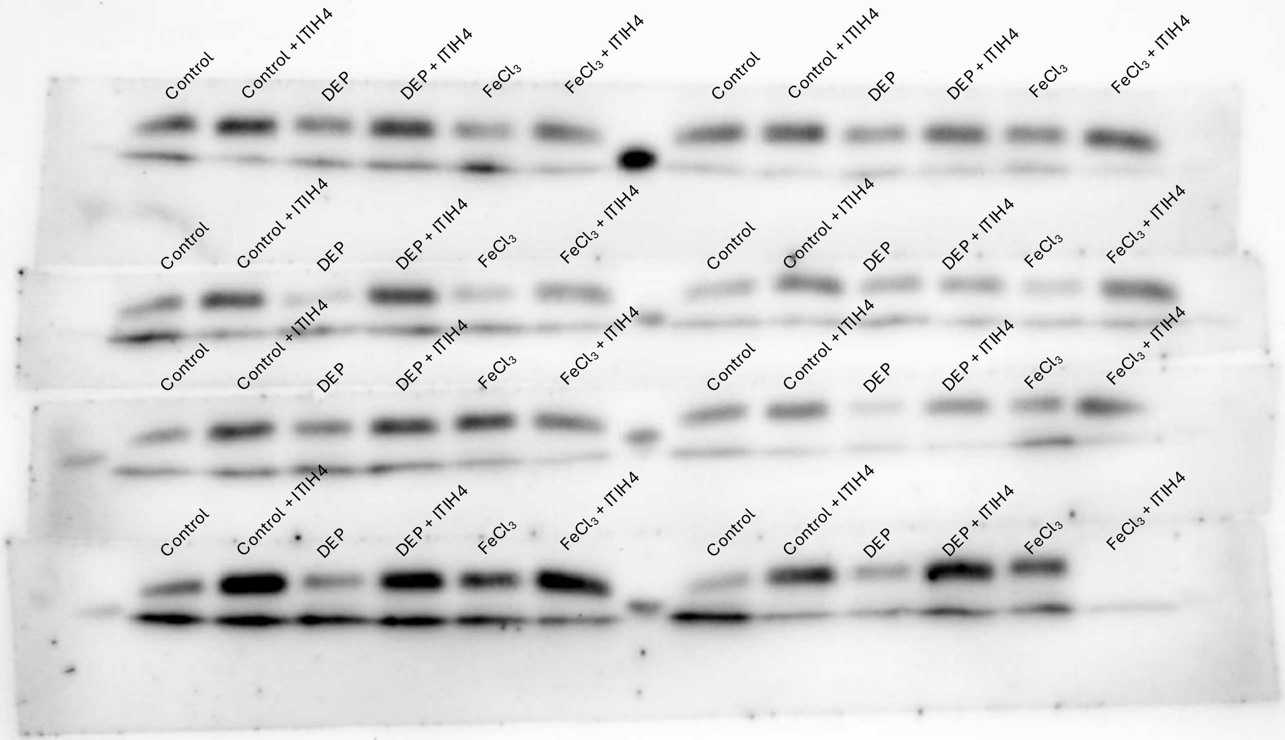

LC3B I 16kDA
LC3B II 14 kDA

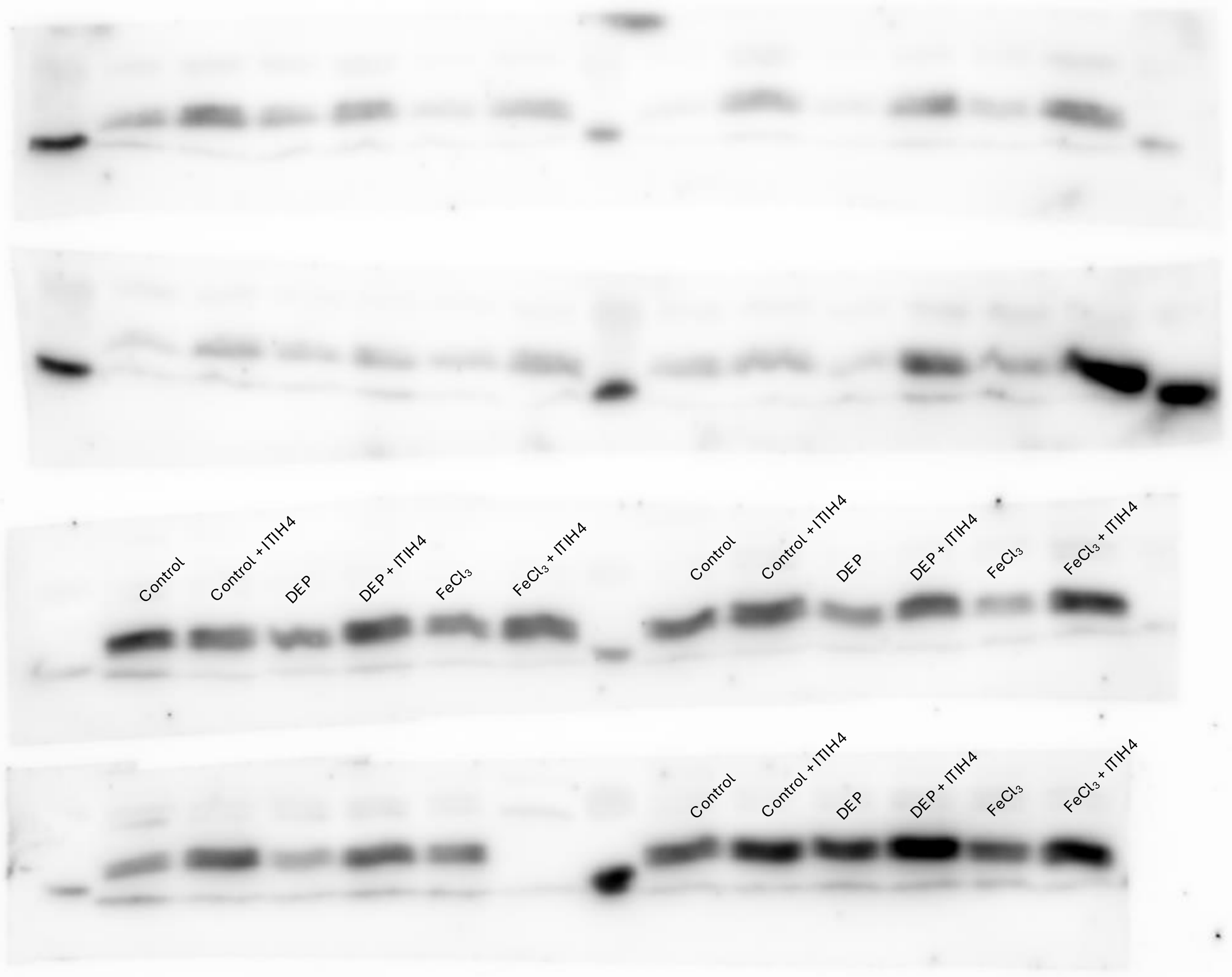

ITIH4
120 kDA

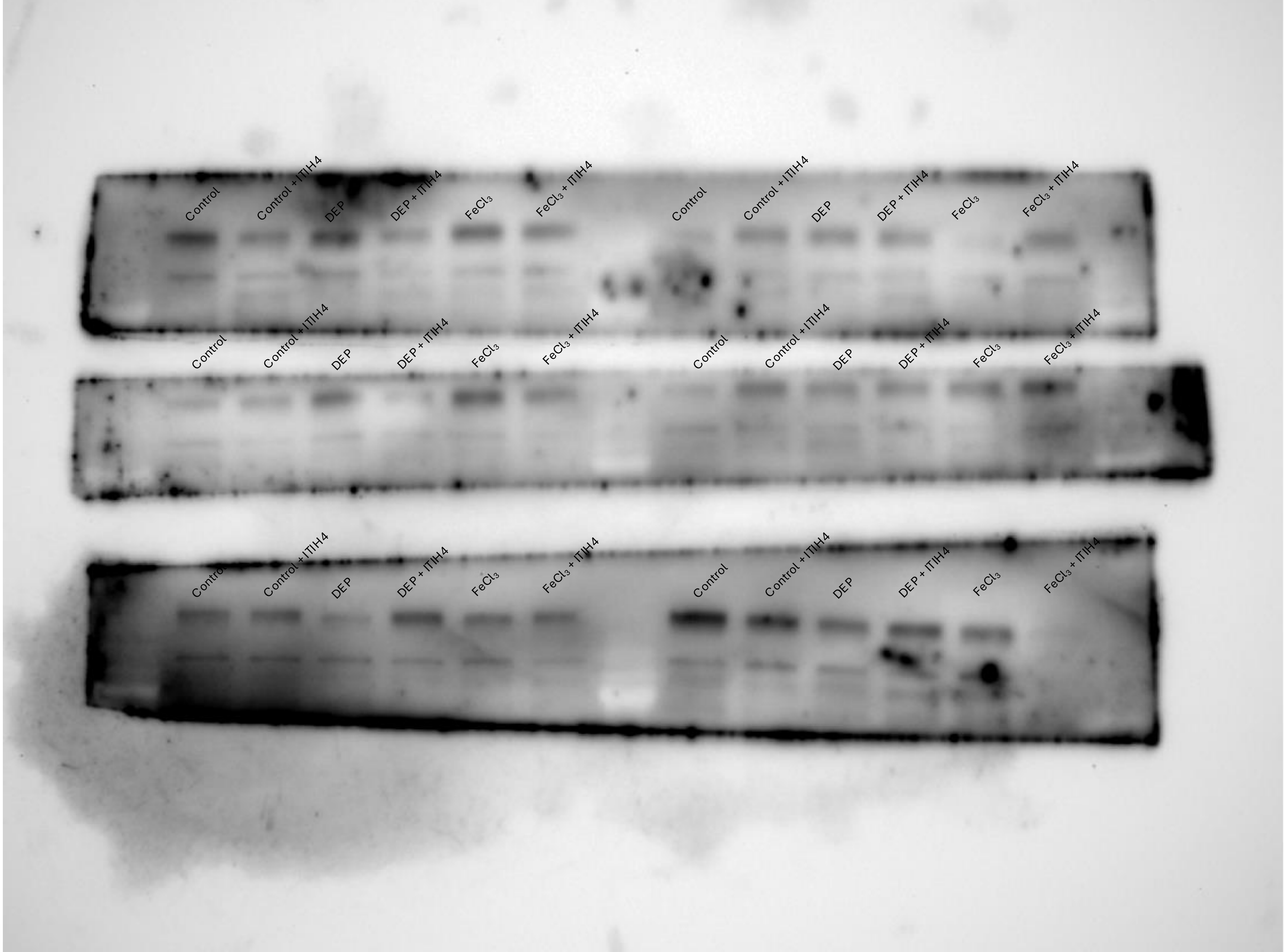

ITIH4
120 kDA

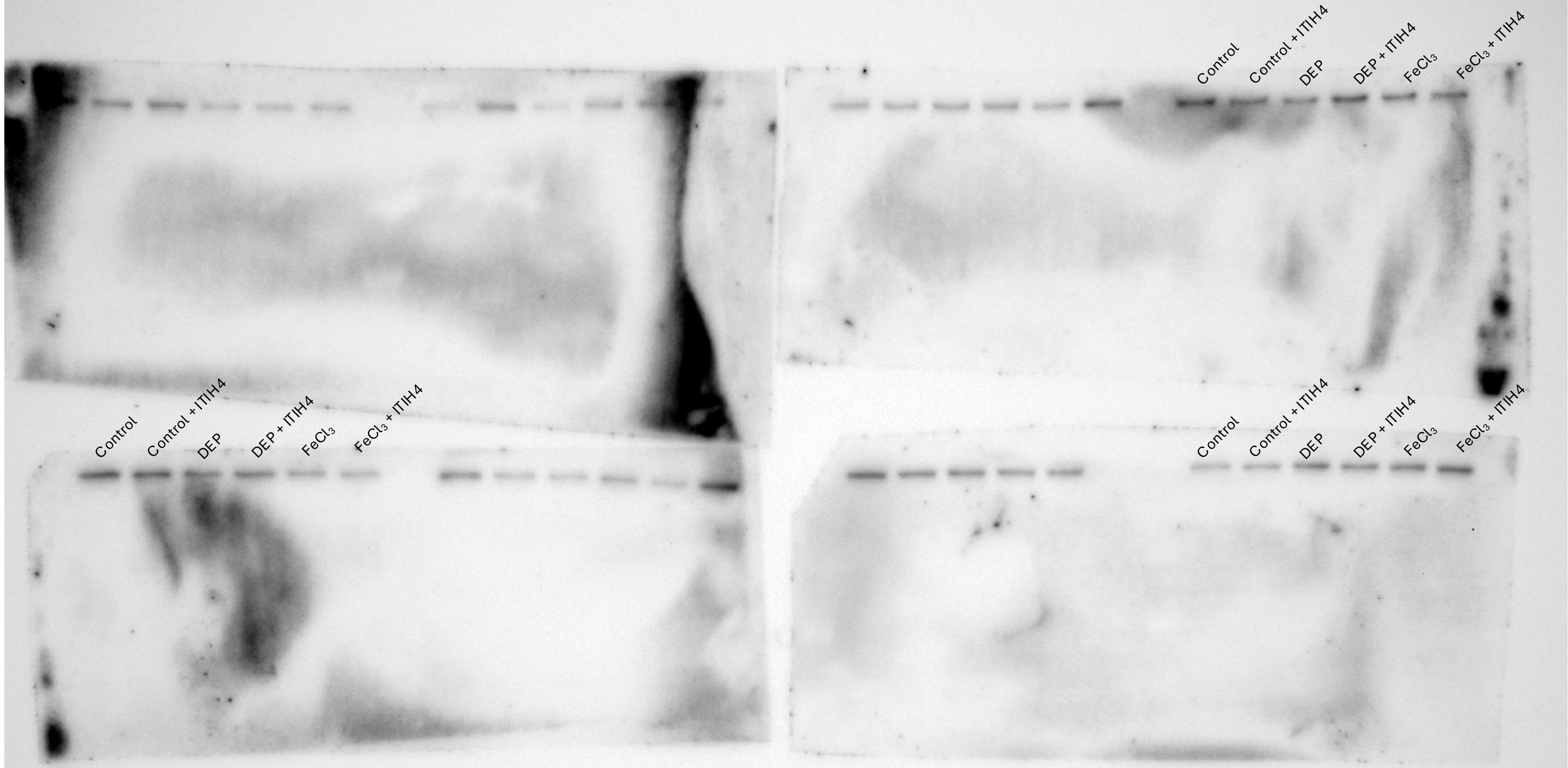

β-actin
45kDA

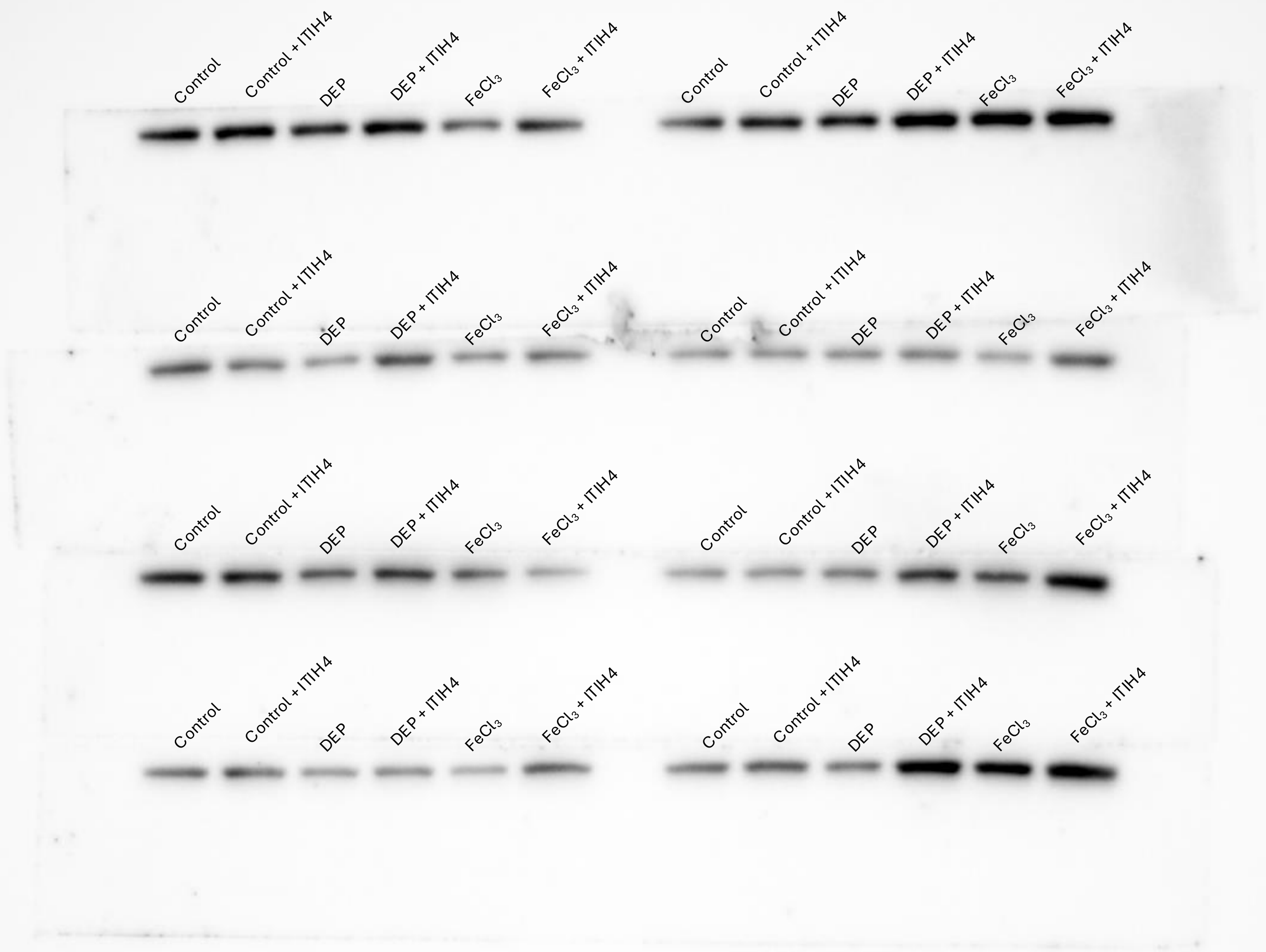

β-actin
45kDA

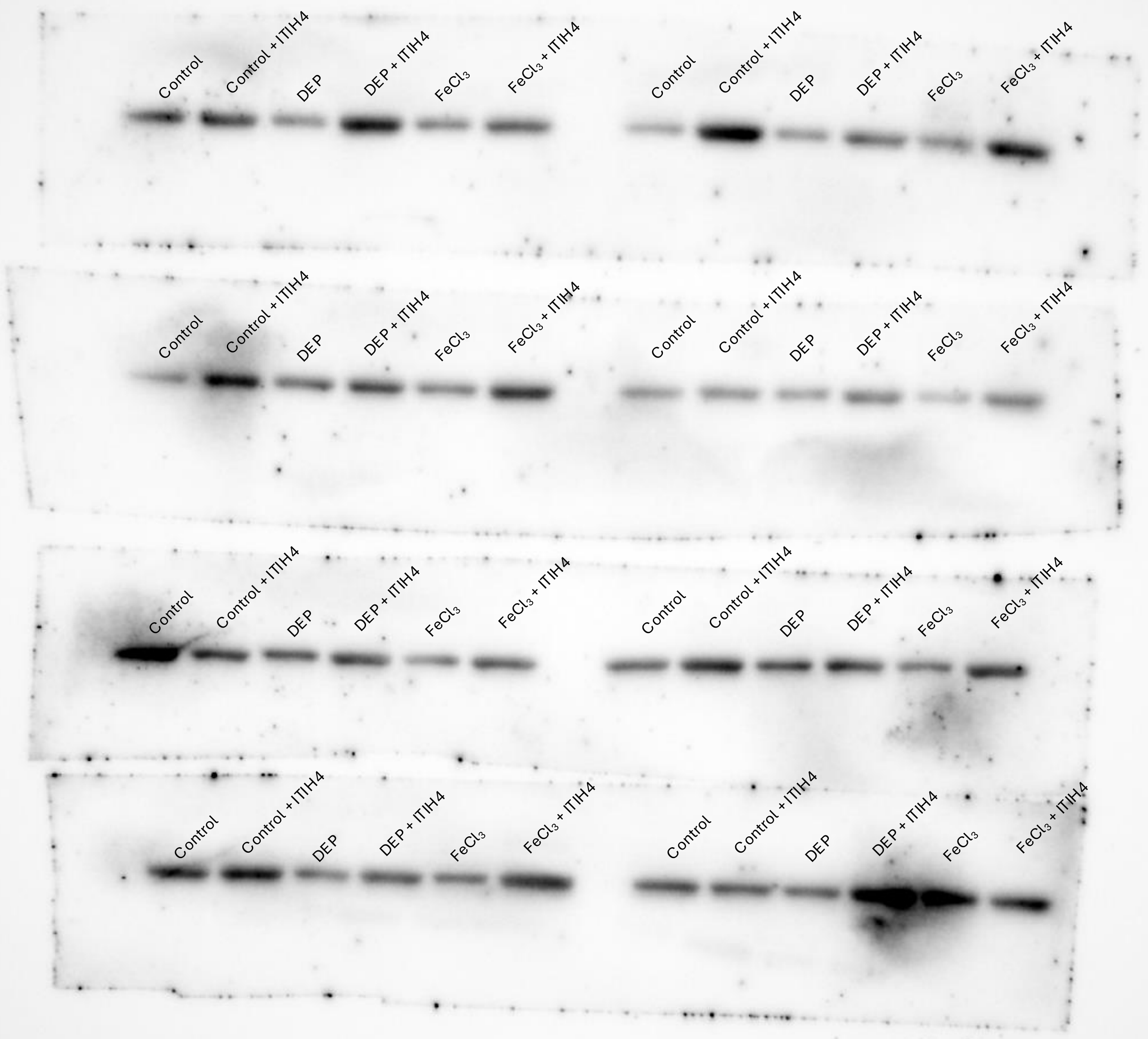

β-actin
45kDA

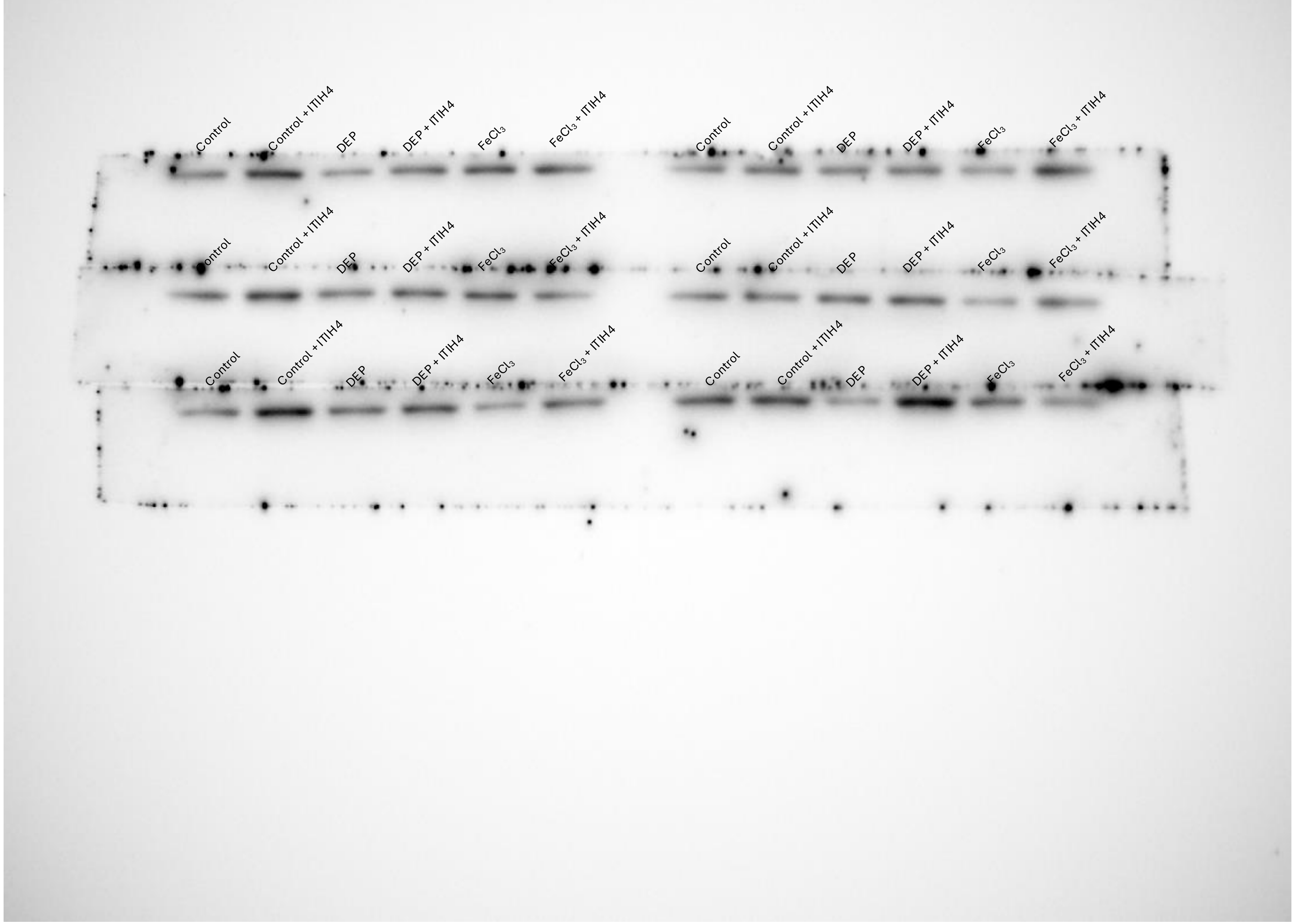

Supplement: Supplementary file 2 — Supplementary Material 2. [file 12931_2025_3256_MOESM2_ESM.pdf]
